# Modulation of virus-induced NF-κB signaling by NEMO coiled coil mimics

**DOI:** 10.1038/s41467-020-15576-3

**Published:** 2020-04-14

**Authors:** Jouliana Sadek, Michael G. Wuo, David Rooklin, Arthur Hauenstein, Seong Ho Hong, Archana Gautam, Hao Wu, Yingkai Zhang, Ethel Cesarman, Paramjit S. Arora

**Affiliations:** 1 Department of Pathology and Laboratory Medicine, Weill Cornell Medical College, New York, NY 10065 USA; 2 Department of Chemistry, New York University, New York, NY 10003 USA; 3 Department of Biological Chemistry and Molecular Pharmacology, Harvard Medical School, Boston, MA 02115 USA; 4 Icahn School of Medicine at Mount Sinai, New York, NY 10029-5674 USA; 5 NYU-ECNU Center for Computational Chemistry, New York University−Shanghai, 200122 Shanghai, China

**Keywords:** Tumour virus infections, Peptides

## Abstract

Protein-protein interactions featuring intricate binding epitopes remain challenging targets for synthetic inhibitors. Interactions of NEMO, a scaffolding protein central to NF-κB signaling, exemplify this challenge. Various regulators are known to interact with different coiled coil regions of NEMO, but the topological complexity of this protein has limited inhibitor design. We undertook a comprehensive effort to block the interaction between vFLIP, a Kaposi’s sarcoma herpesviral oncoprotein, and NEMO using small molecule screening and rational design. Our efforts reveal that a tertiary protein structure mimic of NEMO is necessary for potent inhibition. The rationally designed mimic engages vFLIP directly causing complex disruption, protein degradation and suppression of NF-κB signaling in primary effusion lymphoma (PEL). NEMO mimic treatment induces cell death and delays tumor growth in a PEL xenograft model. Our studies with this inhibitor reveal the critical nexus of signaling complex stability in the regulation of NF-κB by a viral oncoprotein.

## Abstract (web summary)

NF-κB signalling involves the scaffold protein NEMO, which can be bound by the oncoprotein vFLIP to promote cell survival and oncogenic transformation. Here the authors rationally engineer a tertiary protein mimic of NEMO to disrupt the vFLIP-NEMO interaction to induce cell death.

## Introduction

The NF-κB essential modulator (NEMO or IKKγ) serves as a key fulcrum in the NF-κB pathway by relaying upstream signals to the IKK complex catalytic subunits through its elongated coiled coil motif^1^. NEMO is hijacked by various external factors, including viral oncoproteins^2–4^ to initiate aberrant signaling; however, the topological complexity of the NEMO-mediated protein-protein interactions (PPIs) has limited discovery of inhibitors. The challenge of disrupting intracellular tertiary structure mediated protein-protein interactions is well-appreciated^5–7^. While several examples of synthetic inhibitors of secondary structure-mediated protein interfaces have now been described^8–10^, it has been difficult to develop cell-permeable ligands that mimic the complex epitopes of tertiary structures such as those involving NEMO^6^. To address this challenge, we created a synthetic coiled coil mimic of NEMO to engage vFLIP, the Kaposi’s sarcoma herpesvirus (KSHV) viral homolog of CFLAR (or cFLIP)^11,12^. KSHV causes AIDS-associated malignancies, namely primary effusion lymphoma (PEL) and Kaposi sarcoma (KS)^13,14^. We show that the NEMO mimic modulates NF-κB signaling in infected cells and delays tumor growth in a PEL xenograft model. We tested several strategies, including screening with small molecule libraries and an α-helical secondary structure mimic of NEMO, to inhibit NEMO-vFLIP complex formation but found that only an optimized helical tertiary structure mimic of NEMO provides the requisite specificity and potency in modulating cellular signaling.

**Fig. 1:**
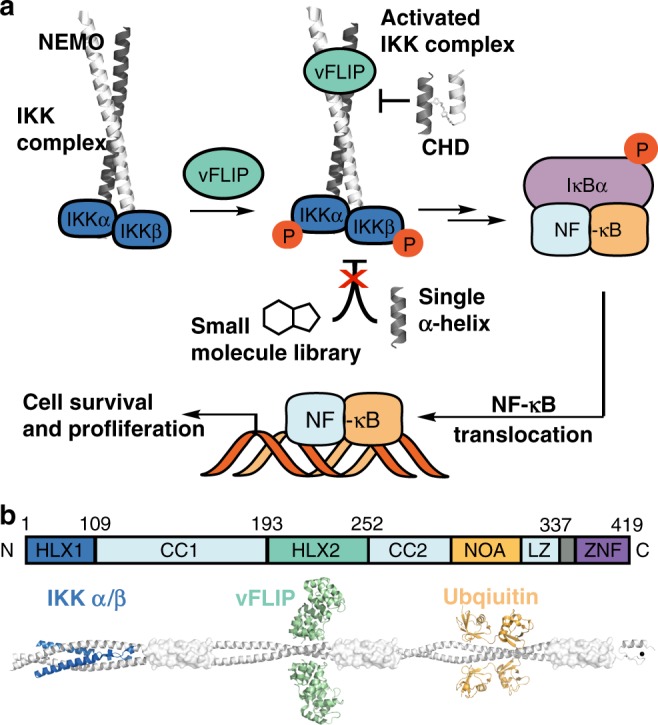
Overview of vFLIP-mediated activation of NF-κB signaling pathway. **a** Binding of vFLIP, a viral oncoprotein, to NEMO activates NF-κB signaling. NEMO adopts a helical coiled coil motif to bind to vFLIP (PDB Code 3CL3). We explored small molecule libraries, stabilized α-helices, and crosslinked helix dimers (CHDs) to inhibit NEMO–vFLIP complex formation. Potent inhibition required a CHD motif that captured critical contacts from both helices of NEMO coiled coil. **b** Cartoon depicts various binding partners of NEMO responsible for activating and repressing NF-κB. vFLIP shown here in green binds the second helical domain (HLX2) downstream of the IKKα/β binding region.

Viral oncogenesis provides an attractive opportunity to develop specific inhibitors of viral oncoproteins that promote tumor survival with less cellular toxicity^15^. Current anti-herpes viral therapies target lytic virus, and are ineffective against latently infected tumor cells^16^. Previous findings indicate that vFLIP is a latent viral oncoprotein that can serve as a potential therapeutic target^17–19^. Transgenic expression of vFLIP induces tumors of B cell origin in mice. vFLIP inhibits apoptosis and autophagy, and can constitutively activate the NF-κBpathway by directly binding to NEMO^17,19–24^, which serves as a hub for binding of different binding partners including IKKα/β subunits (Fig. 1)^3,4,23–25^. The vFLIP/NEMO interaction locks IKK kinase complex into an active phosphorylated state resulting in the downstream activation of genes involved in survival and inhibition of apoptosis^12,24,25^.

A genome-wide CRISPR/CAS9 knockout screen identified cellular genes that are essential for PEL survival, including CFLAR (c-FLIP) and IRF4^26^, and reported that individual genes of the NF-κB pathway are nonessential but rather act as fitness genes in a subset of PEL cell lines. While this approach is powerful at highlighting essential cellular genes, single knock-out studies underestimate compensatory mechanisms and viral factors that contribute to PEL survival. Earlier reports indicated that siRNA-mediated inhibition of vFLIP can significantly decrease NF-κB activity and induce apoptosis^27,28^ and that NEMO is essential for modulating vFLIP-induced gene expression and activating NF-κB and IRF signaling^24,29^. Prior attempts to inhibit the NF-κB pathway to treat PEL include BAY11-7082, an inhibitor of IkBα phosphorylation and the HSP90 inhibitors PU-H71 and BIIB021^30,31^. These studies demonstrate that modulating NF-κB activity can limit PEL progression; however, off-target host specificity has limited their effectiveness as viral directed therapies. A recent report indicated vFLIP essentiality in conferring resistance to apoptosis^19^ and that a single helical IKKγ mimetic can compete the complex and sensitize PEL cells to killing, but only when combined with etoposide (ETO) or TNF-α treatment. These studies support our findings that mimics of single helix strands of a two-stranded NEMO coiled coil do not offer the required affinity to disrupt the target coiled coil complex as single agents, and point to a need for a general strategy for disrupting interactions mediated by helical tertiary structures.

Herein, we describe the rational engineering of an HLX2 domain mimic of NEMO that disrupts NEMO-vFLIP complex as a single agent, thus leading to cell death. Our studies provide unequivocal demonstration that this interaction is essential for the stability of vFLIP in this complex thereby activating NF-κB, an important pro-tumorigenic transcription factor. The viral mechanisms of oncogenesis revealed by our studies suggest an intricate relationship between proteostasis and the NF-κB signalosome.

## Results

### Design and evaluation of NEMO coiled coil mimics

We employed a target-based approach to identify inhibitors of the vFLIP-NEMO interaction^32^. We began by developing a high-throughput Time-Resolved Fluorescence Resonance Energy Transfer (TR-FRET) assay to screen for small molecule vFLIP inhibitors (Supplementary Fig. 1). This in vitro binding assay uses recombinant vFLIP and NEMO fusion proteins bound to donor and acceptor fluorophores, respectively. The assay was sensitive and robust with a Z’ value of 0.92 and is well-suited for high throughput screening of compound libraries. We screened a library of approximately 40,000 diverse and drug-like compounds and identified 20 hits with >40% inhibition which were validated with dose response curves (Supplementary Table 1). Of these compounds, nine with IC_50_ < 65 μM were independently confirmed in the TR-FRET assay. Cytotoxicity of these hits was assayed using CellTiter Glo viability assay. Six of these compounds were active with LC_50_ between 293 nM and 62 μM in KSHV-infected cell lines. However, none of the hits displayed toxicity specific to PEL cells (BC-3), indicating nonspecific results related to vFLIP-independent mechanisms (Supplementary Table 2).

**Fig. 2:**
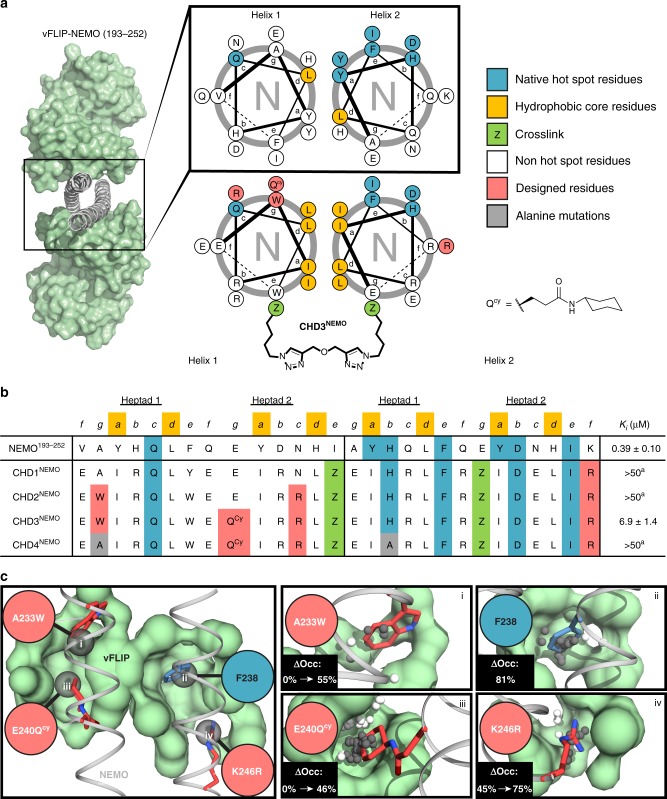
Rational design of inhibitors of the NEMO-vFLIP interaction. **a** Helical wheel diagram depicting native (top) NEMO coiled coil and optimized (bottom) sequences. A crosslinker is placed at the e and g positions to constrain the dimer. **b** Peptide sequences of designed compounds and their respective inhibitory constants from the TR-FRET assay. Superscript ‘a’ denotes incomplete dissociation of the NEMO-vFLIP complex observed at 100 μM concentrations (Fig. 3a). **c** Cartoon depicts four high-ranking pockets identified by AlphaSpace and corresponding residues from **CHD3**^**NEMO**^. The changes in %pocket occupancy as a result of three mutations are highlighted.

We next turned to a rational design approach to develop specific NEMO/vFLIP inhibitors. High resolution structures and computational alanine scanning^33^ reveal residual contact from both helices of the NEMO parallel coiled coil; however, key hot spot residues reside primarily on *Helix 2* of NEMO (Tyr234, His235, Phe238, Tyr241, Asp242, Ile245) as depicted in its helical wheel diagram (Fig. 2a)^12^. To mimic the presentation of these hot spot residues, we designed linear and constrained peptides that span the vFLIP binding region of NEMO (residues 232–245). We utilized the hydrogen bond surrogate (HBS) strategy to lock the peptide into the helical conformation^34,35^. Unfortunately, neither the unconstrained (**NEMO**^232–245^) or the HBS (**HBS**^**NEMO**^) peptide inhibited the vFLIP–NEMO complex formation in the TR-FRET assay at 100 μM concentration (Supplementary Fig. 2). At higher peptide concentrations, observable peptide aggregation was observed. The low solubility of NEMO peptides that mimic a single strand has also been observed in other studies with the side-chain stapled peptides^19^.

Although one helix of the NEMO coiled coil engages vFLIP with stronger contacts, as suggested by the crystal structure of the complex^12^ and our computational analysis, we hypothesized that *Helix 2* of NEMO is unable to properly orient itself on the vFLIP binding surface without the coiled coil partner. Based on this premise, we aimed to develop a NEMO coiled-coil mimic to modulate the target interaction. The coiled coil motif is not stable in short peptide sequences because short sequences do not offer enough interhelical contacts to enable formation of the dimeric assembly. We recently reported a strategy to generate synthetic coiled coil mimics, termed Crosslinked Helix Dimers (CHDs), by judiciously replacing an interhelical salt bridge with a covalent bond and sculpting optimal knob-into-hole helix packing^36,37^. The optimized salt bridge surrogate and helix packing are required for high conformational stability. A parallel coiled coil mimic is optimal with *e-g’* position azidolysine residues crosslinked with propargyl ether using copper-catalyzed azide-alkyne cycloaddition^38^.

We attempted to develop a NEMO coiled coil mimic with native residues; however, the native sequence features nonoptimal knob-into-hole helix packing (Fig. 2a)^39^. To optimize conformational stability, Tyr234 and Tyr241 were replaced with isoleucine residues and His244 mutated to leucine. Unfortunately, both tyrosine residues make critical contacts with the vFLIP surface (Supplementary Table 3), which significantly diminished the binding affinity in the designed CHDs.

The failure of the CHDs bearing wild-type residues to provide potent inhibition prompted us to optimize the NEMO coiled coil with noncanonical residues to overcome the loss of the two tyrosine hot spot residues. We utilized AlphaSpace to obtain fragment-centric topographical mapping of protein surfaces to identify underutilized pockets in PPIs^40^ and enhance target engagement^41^ by introducing non-canonical residues. We discovered several key pockets on the vFLIP surface that could be targeted using natural and non-natural amino acids displayed from the coiled-coil scaffold. AlphaSpace provides a pocket occupation score, which can be used as a guide to predict optimal noncanonical residues.

We used an iterative process of design, synthesis, biophysical and biological characterization to optimize NEMO coiled coil mimics. The sequences and TR-FRET derived inhibitory constants (K_i_) for each peptide are listed in Fig. 2b. Briefly, AlphaSpace predicted that incorporation of Lys246 to Arg246 mutation would increase occupancy of *pocket iv* from 45 to 75% (Fig. 2c). **CHD1**^**NEMO**^ was synthesized to evaluate the effect of this modification while maintaining essential hot spot residues such as F238, which has a native pocket occupancy of 81% (pocket *ii*). In order to impart optimal interhelical packing, the interhelix residue Tyr234, which is a key hot spot residue, was mutated to Leu234. This mutation was compensated for by converting the neighboring Ala233 residue on *Helix 1* to tryptophan. Tryptophan is predicted to occupy 55% of *pocket i*. The native alanine residue did not engage this pocket. **CHD2**^**NEMO**^ contains this change along with N243R and K246R in *Helix 1* and *Helix 2*, respectively. The highest-ranking underutilized pocket is detected near *Helix 1* but is located too far from the NEMO backbone to be contacted by canonical amino acids. We derivatized Glu240 of *Helix 1* with a cyclohexylamine group to obtain glutamine cyclohexyl amide (Q^Cy^), and this substitution is predicted to provide 46% pocket occupancy (pocket *iii*) (Fig. 2c). **CHD3**^**NEMO**^ combines the earlier mutations with Q^Cy^. AlphaSpace predicts that **CHD3**^**NEMO**^ would be a high affinity ligand for vFLIP (Supplementary Table 5). We also designed and synthesized **CHD4**^**NEMO**^ as a specificity control for **CHD3**^**NEMO**^. **CHD4**^**NEMO**^ shares structural and sequence similarity with **CHD3**^**NEMO**^ but contains alanine in place of one key hot spot residue on each helix at positions 233 of *Helix 1* and 235 of *Helix 2* (Fig. 2b). We predicted **CHD4**^**NEMO**^ would have a diminished effect when compared to **CHD3**^**NEMO**^, but not be completely ineffective. **CHD2**^**NEMO**^ serves as another control that lacks the hydrophobic side chain from Q^Cy^ and allows us to probe the influence of this side chain on the cellular uptake and potency of the lead inhibitor **CHD3**^**NEMO**^.

### CHD3^NEMO^ is conformationally and proteolytically stable and enters live cells

**Fig. 3:**
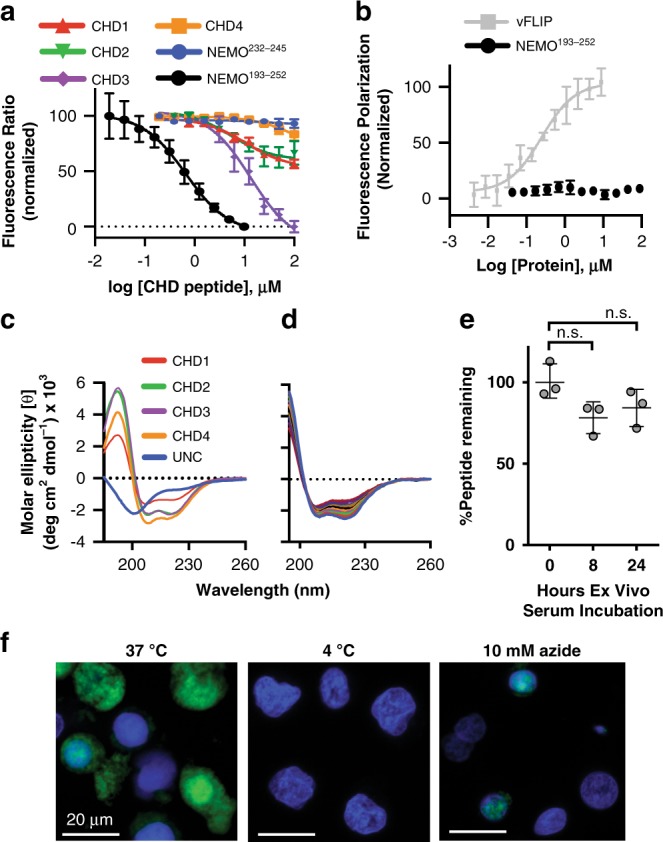
Biophysical characterization, stability and cellular uptake of NEMO mimics. **a** The potential of NEMO mimics to inhibit vFLIP-NEMO interaction was evaluated in a TR-FRET assay by monitoring the fluorescence emission of the acceptor. The data represents the mean ± SD of two independent experiments (*n* = 2) each performed with at least three replicates. These studies illustrated the potency of the optimized derivative **CHD3**^**NEMO**^. **b** The specific association of the FITC-derivatized **CHD3**^**NEMO**^ with vFLIP over full length NEMO was further probed in a fluorescence polarization assay. The data represents the mean ± SD of a single experiment performed in triplicates. This experiment was repeated twice (*n* = 2) with similar results. **c** The conformation of the coiled coil mimics was investigated by circular dichroism spectroscopy in aqueous buffer. **d** The conformational stability of **CHD3**^**NEMO**^ was further evaluated in a thermal denaturation study. The circular dichroism spectra were collected at regular intervals between 5–95 °C. **e** **CHD3**^**NEMO**^ was found to resist serum proteases. Peptide degradation was probed over 24 h by HPLC. This data represents mean ± SD (*n* = 2) **f** Cellular uptake of FITC-labeled **CHD3**^**NEMO**^ into live BC-1 cells. Cells were visualized by fluorescence microscopy after 1 h incubation. Effect of temperature and 10 mM sodium azide on the cellular uptake of the NEMO mimic was also explored. Hoechst stain was used to detect the nuclei. Representative images are shown from 2 independent experiments (*n* = 2). Scale bar = 20 μm.

The in vitro binding results support the computational predictions. **CHD1**^**NEMO**^, **CHD2**^**NEMO**^, and **CHD4**^**NEMO**^ provided only partial inhibition of the vFLIP–NEMO complex at 100 μM concentrations (Fig. 3a); whereas **CHD3**^**NEMO**^ led to robust inhibition of vFLIP-NEMO complex formation (*K*_i_ = 6.9 ± 1.4 μM). Under similar conditions, the wild-type NEMO coiled coil (NEMO^193–252^) modulates the complex with a submicromolar inhibitory constant (*K*_i_ = 0.39 ± 0.10 μM). We also developed a fluorescence polarization binding assay to gauge the affinity of fluorescein-labeled derivative of **CHD3**^**NEMO**^ for vFLIP. In this direct binding assay, the *K*_d_ of **FITC-CHD3**^**NEMO**^ for vFLIP was calculated to be 230 ± 100 nM (Fig. 3b). This NEMO mimic does not bind wild-type NEMO coiled coil providing confidence that changes in the TR-FRET signal resulted from binding of the CHDs to vFLIP and not non-specific binding to NEMO.

Together the three unoptimized CHDs (**CHD1**^**NEMO**^, **CHD2**^**NEMO**^, and **CHD4**^**NEMO**^) highlight the role of the native and noncanonical hot spot residues in affording a potent designed ligand for vFLIP. We obtained circular dichroism spectra (CD) of the CHDs under aqueous buffers to gauge the impact of conformational stability on binding. Each mimetic shows a convincing alpha helical signature as demonstrated by local minima at 222 nm and 208 nm typical of coiled coils (Fig. 3c). Each CHD is highly helical, with the exception of **CHD1**^**NEMO**^. We hypothesize that mutation of Asn243 to arginine results in an additional salt bridge aiding the helical stability of **CHD2-4**. Coiled coils tend to multimerize or aggregate into larger assemblies. We utilized high performance size exclusion chromatography with appropriate protein molecular weight markers to determine if the CD signature of the mimics reflects constrained dimers, as designed, or higher order assemblies. This assay confirmed that **CHD3**^**NEMO**^ does not aggregate in aqueous buffers (Supplementary Fig. 3).

Next, we analyzed the conformational stability of **CHD3**^**NEMO**^ by monitoring changes in its circular dichroism spectrum as a function of temperature (Fig. 3d). We found the NEMO mimic to retain a significant proportion of helix percentage at high temperatures. We also probed the ability of **CHD3**^**NEMO**^ to withstand serum proteases and observed it was highly resistant to degradation whereby roughly 80% of the initial peptide remained intact after 24 h of serum incubation (Fig. 3e). Together the thermal denaturation and serum stability assays revealed the high conformational and proteolytic stability of **CHD3**^**NEMO**^.

Peptides often do not enter cells without exogenous delivery strategies or peptide modifications^42–44^. We tested the ability of the fluorescein-derivatized coiled-coil mimic, **FITC-CHD3**^**NEMO**^ to enter PEL cells using live cell confocal microscopy. We found that this compound enters BC-1 cells; although not all cells had observable amounts of the fluorescein signal. Hoechst nuclear stain was employed in combination with **FITC-CHD3**^**NEMO**^ and is shown as an overlay (Fig. 3f). To validate the design strategy and demonstrate that cellular entry was not just an inherent property of the **CHD3**^**NEMO**^ peptide, we tested the uptake of the control peptide **FITC-CHD2**^**NEMO**^ that lacks Q^cy^ noncanonical residue (Supplementary Fig. 4) and observed a similar immunofluorescence staining suggesting that the crosslinked NEMO derivatives are cell-permeable independent of the minor sequence differences. In order to probe the cellular entry mechanism of **FITC-CHD3**^**NEMO**^, we performed cellular uptake experiments at 4 °C, as well as with sodium azide poisoning to reduce ATP-mediated active transport mechanisms without affecting overall cellular viability (Supplementary Fig. 13)^45^. The reduction in cellular uptake at cold temperatures and in the presence of sodium azide suggests an active transport mechanism for **CHD3**^**NEMO**^ (Fig. 3f);  however, a detailed analysis of the transport mechanism remains to be undertaken. We hypothesize that the arginine-rich nature of the crosslinked peptides is aiding their uptake^46^.

### CHD3^NEMO^ inhibits vFLIP-mediated NF-κB activation in a dose-dependent manner

**Fig. 4:**
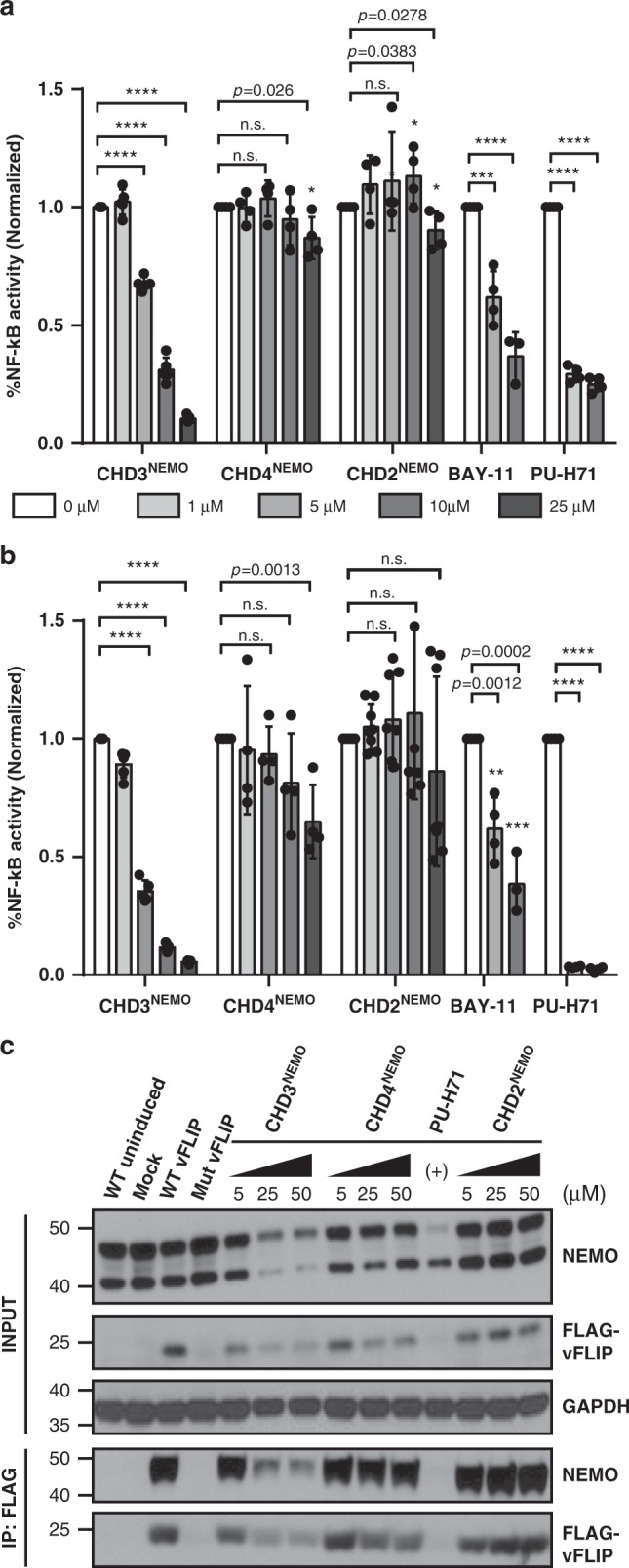
CHD3^NEMO^ suppresses vFLIP-mediated NF-κB transcriptional activity and disrupts NEMO-vFLIP complex formation. **a** **CHD3**^**NEMO**^, but not control peptide **CHD2**^**NEMO**^ or **CHD4**^**NEMO**^, inhibits NF-κB transcriptional activity significantly in BC-3 NF-κB-luc PEL cells in a dose-dependent manner at (**a**) *t* = 5 h and (**b**) *t* = 24 h post-treatment. BC-3 reporter cell line was treated with increasing concentrations of the different NEMO mimetics or in the presence of the NF-κB inhibitor Bay 11-7082 or HSP90 inhibitor PU-H71. Luciferase assays were performed at the indicated time points. Statistical analysis was performed using two-tailed unpaired t-test comparing treated samples to DMSO control (****p ≤ 0.0001, ***p ≤ 0.001, **p ≤ 0.01, *p ≤ 0.05 and non-significant *p* > 0.05). The data represents mean ± SEM of two independent experiments (*n* = 2) performed in triplicates. **c** **CHD3**^**NEMO**^ disrupts vFLIP/NEMO interaction in live cells. Co-immunoprecipiation with an anti-FLAG M2 beads was performed using a vFLIP–FLAG doxycycline-inducible Namalwa cell line. Results show a dose-dependent reduction in the levels of interacting NEMO upon treating cells with the vFLIP inhibitor **CHD3**^**NEMO**^ but not with **CHD2**^**NEMO**^ or **CHD4**^**NEMO**^ peptide. WT uninduced: Namalwa WT vFLIP stable cell line without induction with doxycycline; Mock: parental Namalwa cell line; Mut vFLIP: Namalwa stable cell line carrying vFLIP NF-κB dead mutant that lacks the ability to bind to NEMO. Mut vFLIP and WT vFLIP cell lines were treated with doxycycline to induce vFLIP expression 24 h prior to treating WT vFLIP cell line with DMSO or the different NEMO mimetics.

To probe the potential of NEMO mimetics to specifically engage vFLIP and modulate NF-κB transcriptional activity in PEL cells, we treated the BC-3 NF-κB luciferase reporter cell line (BC-3-NF-κB-luc) with increasing doses of the **CHD2**^**NEMO**^, **CHD3**^**NEMO**^ or **CHD4**^**NEMO**^ peptides (Fig. 4). Bay 11-7082 (inhibitor of IκBα phosphorylation) and PU-H71 (HSP90 inhibitor) were used as positive controls in these studies. HSP90 is known to chaperone both vFLIP and NEMO and treatment with its inhibitor PU-H71 is known to destabilize both proteins^30^. We found that **CHD3**^**NEMO**^ significantly inhibited NF-κB transcriptional activity in a dose-dependent manner both at *t* = 5 h (Fig. 4a) and *t* = 24 h (Fig. 4b) post-treatment in contrast to **CHD2**^**NEMO**^ **and CHD4**^**NEMO**^, which showed minimal activities. Treatment of cells with 10 μΜ **CHD3**^**NEMO**^ for 24 h led to 90 percent suppression of NF-κB activity, whereas the control derivative **CHD2**^**NEMO**^ showed minimal transcriptional downregulation at the same concentration, further demonstrating the importance of critical hot spot residues in engaging vFLIP. **CHD4**^**NEMO**^, which serves as a specificity control, shows approximately 10-fold less inhibition of NF-κB activity in the reporter assay than **CHD3**^**NEMO**^. The effect of **CHD4**^**NEMO**^ only manifests itself at the highest dose and at a later time point.

To further illustrate the effects of **CHD3**^**NEMO**^ on NF-κB activation, we analyzed components of NF-κB signaling in nuclear and cytoplasmic extracts of BC-1 cell line by western blotting. We observed a significant reduction in the levels of phosphorylated p65 and total p65 in the nuclear extracts of cells treated with 25 μM of **CHD3**^**NEMO**^, while these levels remained unaltered in the cytoplasmic extracts (Supplementary Fig. 5a). When we probed for upstream components of IKK signaling, we observed a parallel reduction in the cytoplasmic levels of the phosphorylated IKKα/***β*** at the 25 μM dose (Supplementary Fig. 5b). To demonstrate that the reduction of NF-κB activity upon **CHD3**^**NEMO**^ treatment is specific to vFLIP-NEMO interaction and not due to off target effects, we examined NF-κB signaling in HeLa cells pretreated with increasing doses of **CHD3**^**NEMO**^ followed by TNFα stimulation. We observed a dramatic reduction in the total levels of IkBα only in the TNFα stimulated cells but no changes in the total levels of IKKα, IKK***β***, p65, or NEMO. Importantly, the IkBα degradation observed upon TNFα treatment was not attenuated by **CHD3**^**NEMO**^ treatment (Supplementary Fig. 6), and there was no evidence of **CHD3**^**NEMO**^ inhibiting NF-kB activation in these vFLIP-negative cells.

### CHD3^NEMO^ disrupts vFLIP/NEMO complex formation in lymphoma cells

We next used co-immunoprecipitation studies to investigate whether **CHD3**^**NEMO**^ disrupted vFLIP/NEMO complex formation. We used a Namalwa Burkitt lymphoma cell line, which was stably transfected with the tetracycline-inducible FLAG-tagged wildtype (WT vFLIP) or mutant (Mut vFLIP) plasmids. We used Mut vFLIP as a positive control since it is an NF-κB dead vFLIP mutant that cannot engage intracellular NEMO as indicated by the absence of NEMO in the immunoprecipitated lysates (Fig. 4c). We induced expression of WT vFLIP or Mut vFLIP with doxycycline for 24 h. Next, we dosed cells expressing wild-type vFLIP with DMSO or increasing concentrations of **CHD3**^**NEMO**^, **CHD2**^**NEMO**^, **or CHD4**^**NEMO**^ peptides for additional 24 h. Cells were lysed and immunoprecipitated using anti-FLAG antibody beads followed by immunoblot analysis using NEMO antibody (Fig. 4c). Treatment of cells with increasing concentrations of **CHD3**^**NEMO**^ resulted in a decrease of both vFLIP and NEMO protein levels, as well as a dose-dependent disruption of the vFLIP/NEMO complex indicated by the reduced levels of NEMO that are pulled down by the FLAG beads. As expected, the target complex was not disrupted in cells treated with negative controls **CHD2**^**NEMO**^ and to a lesser extent with **CHD4**^**NEMO**^, which engages the target vFLIP and inhibits NF-κB activity only at higher concentrations.

The reduction of both vFLIP and NEMO protein levels in Namalwa cells treated with **CHD3**^**NEMO**^ (Fig. 4c) suggested that disruption of this interaction destabilizes both proteins. This result is consistent with the effect of HSP90 inhibitor PU-H71, which is known to destabilize both proteins leading to reduced interaction^30^. However, HSP90 is a chaperone protein which impacts the stability of a multitude of cellular proteins. The NEMO mimic provides a direct reagent to probe if vFLIP binding to NEMO is essential for the stability of the NF-κB signalosome. We observed a reduction in vFLIP levels in BC-1 PEL lysates upon treatment with 50 μM **CHD3**^**NEMO**^ (Supplementary Fig. 7). This decrease is also observed when the lysate is subjected to immunoprecipitation with the NEMO antibody but not in the IgG isotype control (Supplementary Fig. 8). Immunoblot of vFLIP inducible Namalwa line protein extracts showed dose-dependent decrease in vFLIP and NEMO levels upon treatment with increasing concentrations of **CHD3**^**NEMO**^ compared to slight reduction in NEMO levels in the parental Namalwa cell line at 50 μM dose. The latter results suggest additional effects of **CHD3**^**NEMO**^ on other NEMO interactors at higher concentrations (Supplementary Fig. 9). In continuing studies, we are preparing analogs of **CHD3**^**NEMO**^ and other segments of NEMO, to identify their cellular partners using chemoproteomics analyses. The results of these studies, which will be reported in due course, will allow dissection of the NEMO interactome on a global scale.

To determine if genetic mutations that abolish vFLIP’s ability to bind NEMO (mut vFLIP) recapitulate the phenotypic effect observed upon the pharmacological inhibition of the complex using **CHD3**^**NEMO**^, we looked at vFLIP levels in Namalwa WT and Namalwa Mut vFLIP cell lines. Interestingly, there were low levels of NEMO and significantly reduced levels of vFLIP protein in the vFLIP NF-κB dead mutant control suggesting that NEMO binding is essential for vFLIP protein expression – consistent with earlier observations^12^. Treatment of Namalwa Mut vFLIP cell line with the proteasomal inhibitor MG132 stabilized vFLIP protein and increased NEMO levels within 2 h of treatment (Supplementary Fig. 10a). This result is in agreement with earlier reports showing that vFLIP degradation is mediated via the ubiquitin-proteasomal pathway^47,48^. To determine whether mutation in NEMO binding affected vFLIP stability, we treated cells with cycloheximide to block protein synthesis and found that vFLIP mutant has a shorter half-life compared to WT vFLIP (Supplementary Fig. 10b). The above studies suggest a model in which binding of vFLIP to NEMO fortifies the complex; disruption of this complex using **CHD3**^**NEMO**^ leads to destabilization of both proteins and their subsequent proteasomal degradation.

In order to assess whether the designed inhibitor is indeed able to disrupt the complex, we utilized the proteasomal inhibitor, MG132 to prevent vFLIP and NEMO proteasomal degradation and examined binding of vFLIP to NEMO upon **CHD3**^**NEMO**^ treatment. Under these conditions, we detected reduced binding of vFLIP to NEMO and a parallel dose-dependent decrease in the levels of IKKβ, the catalytic subunit that is also a component of the IKK signalosome^3^ as shown in the immunoprecipitated lysates further corroborating the disruption of the complex (Supplementary Fig. 10c). These results strongly support a model where **CHD3**^**NEMO**^ downregulates NF-κB activity by directly inhibiting the vFLIP/NEMO complex, destabilizing the IKK signalosome complex and suppressing vFLIP-induced IKK activation. The studies also support the model that both vFLIP and NEMO protein stability are complex dependent.

### Designed mimetics show selective cytotoxicity towards vFLIP expressing cell lines

**Fig. 5:**
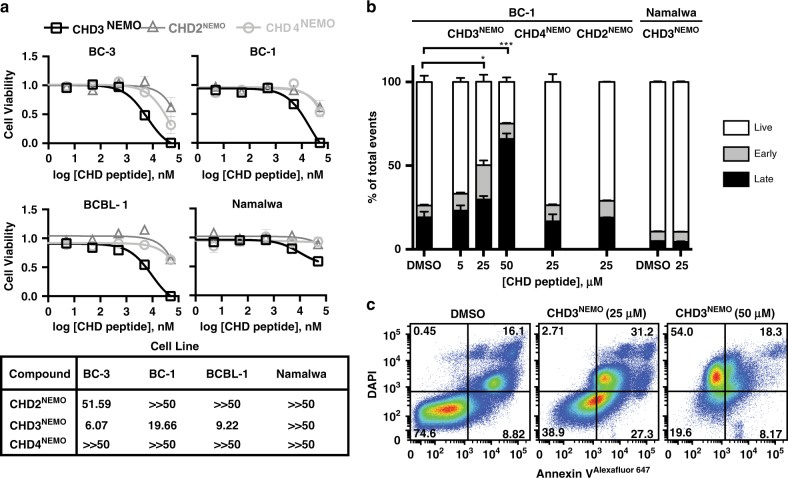
CHD3^NEMO^ induces cell death in vFLIP-expressing PEL cell lines. **a** **CHD3**^**NEMO**^ but not **CHD2**^**NEMO**^ **or CHD4**^**NEMO**^ controls induces cell death in a panel of PEL cell lines. vFLIP (−) Namalwa cell line was used as a control. BC-3, BCBL-1, BC-1, and Namalwa cells were treated with increasing concentrations of **CHD2**^**NEMO**^, **CHD3**^**NEMO**^**, CHD4**^**NEMO**^, and cytotoxicity was assayed using the CellTiter-Glo assay at *t* = 24 h and *t* = 72 h, respectively. Data represents mean ± SEM of (*n* = 3) independent experiments performed in duplicates. The IC_50_s (µM) of the CHDs in the tested cell lines are displayed in the table below the graphs. **b**–**c** Flow cytometry analysis showing that **CHD3**^**NEMO**^ induces apoptosis in vFLIP (+) BC-1 PEL cell line, but not vFLIP (−) Namalwa. **CHD2**^**NEMO**^ **and CHD4**^**NEMO**^ had no effect. BC-1 cells were treated with DMSO, 5 µM, 25 µM or 50 µM of **CHD3**^**NEMO**^ for 48 h. After staining for DAPI and Annexin V, cells were examined using flow cytometry. Results were quantified into percentages of live (Annexin V−, DAPI−), early apoptotic cells (Annexin V+) or late apoptotic cells (Annexin V+/ DAPI + and DAPI+). Results shown are the mean ± SEM of (*n* = 3) independent experiments. Statistical analysis was performed using two-tailed unpaired *t*-test comparing treated samples to DMSO control. DMSO vs 25μM **CHD3**^**NEMO**^ early apoptotic population *p* = 0.0124, DMSO versus 50 μM **CHD3**^**NEMO**^ late apoptotic *p* = 0.0010 (*p ≤ 0.05, ***p ≤ 0.001 respectively). One-way ANOVA analysis was performed comparing 25 μM dose of each of **CHD2**^**NEMO**^, **CHD3**^**NEMO**^, **CHD4**^**NEMO**^ peptides and was found to be significant with *p* = 0.0087, **p ≤ 0.01).

To determine whether disruption of the vFLIP/NEMO complex in PEL cells was accompanied with cell death, we examined cytotoxicity of the different NEMO mimetics in a panel of PEL cell lines (BC-1, BC-2, BC-3, BCBL-1) and Namalwa cells, a Burkitt B-cell lymphoma cell line that does not express vFLIP. **CHD3**^**NEMO**^ significantly decreased the cellular viability of PEL cells as assessed using CellTiter-Glo cell viability assay. The compound showed significant toxicity towards cell lines that expresses high levels of vFLIP protein. All PEL cell lines were more sensitive to **CHD3**^**NEMO**^ treatment (Fig. 5a) than **CHD2**^**NEMO**^ and **CHD4**^**NEMO**^. Similar experiments conducted in vFLIP-negative Namalwa cells showed little to no sensitivity upon treatment with all three peptides 72 h post-treatment. In contrast, both PEL and Namalwa cell lines showed sensitivity to the HSP90 inhibitor PU-71 (Supplementary Table 4). This data are consistent with earlier observations showing vFLIP essentiality in PEL cell lines. Taken together, these data demonstrate that **CHD3**^**NEMO**^ shows selective cytotoxicity in vFLIP expressing cell lines. This is likely a result of modulating its target vFLIP levels and perturbing downstream effectors that contribute to PEL survival.

### CHD3^NEMO^ induces apoptosis in BC-1 PEL cell line

To elucidate the mechanism of **CHD3**^**NEMO**^ induced cell death, we performed Annexin V staining to detect whether PEL cells underwent apoptosis upon **CHD3**^**NEMO**^ treatment (Fig. 5b). We treated BC-1 or Namalwa cells with DMSO or increasing concentrations of **CHD3**^**NEMO**^ and 25 μM doses of **CHD2**^**NEMO**^ or **CHD4**^**NEMO**^ (Fig. 5b). Cells were treated with the NEMO mimics for 48 h, stained with Annexin V and DAPI and analyzed for cell death markers using flow cytometry. We observed a significant increase in early apoptotic cells (Annexin V positive, *p* < 0.05) in BC-1 PEL cells treated with 25 μM **CHD3**^**NEMO**^ compared to DMSO control (Fig. 5c) (Fig. 5b) compared to the same dose of the other NEMO mimetics (ANOVA, **p < 0.01). Cells treated with 50 μM dose showed a significant increase in Annexin V/DAPI double positive and single DAPI positive cells indicating late apoptosis. However, we did not detect any apoptosis upon treating BC-1 cells with 25 μM **CHD2**^**NEMO**^ or **CHD4**^**NEMO**^ or treating the vFLIP (−) control Namalwa cell line with **CHD3**^**NEMO**^. Collectively, these data suggest that **CHD3**^**NEMO**^ treatment promotes apoptosis in vFLIP-expressing PEL cell lines.

### In vivo assessment of CHD3^NEMO^ in a PEL xenograft mouse model

**Fig. 6:**
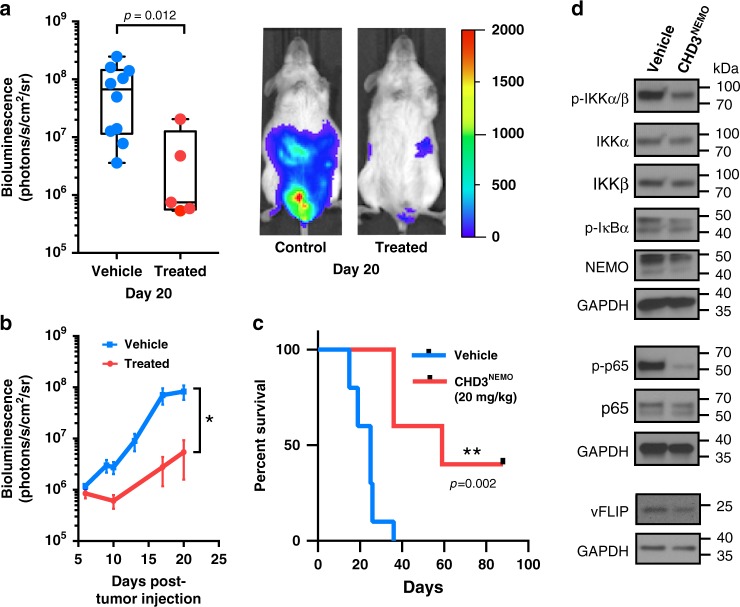
Effect of CHD3^NEMO^ on tumor growth in a traceable reporter BC-3-Luc PEL xenograft mouse model. **a** Box- and Whisker plots of relative tumor burden at day 20 post-engraftment quantified using bioluminescence imaging. Statistical analysis was performed using two-sided Mann Whitney test (*p* = 0.012, *p ≤ 0.05). One representative mice for each group is presented (right). Box and whisker plots represent all individual data points within the /min-max range (vehicle *n* = 10 and treated *n* = 5). **b** Bioluminescence quantitation representing tumor burden (mean ± SEM) after 20 days of tumor engraftment in the vehicle-treated (blue line, *n* = 10) versus **CHD3**^**NEMO**^ treated group (red line, *n* = 5). Statistical analysis was performed using two-sided Mann Whitney test (*p* = 0.012, *p ≤ 0.05). **c** Kaplan-Meier survival analysis showing that mice treated with the **CHD3**^**NEMO**^ peptide (in red) has a survival advantage compared to the control group (in blue). The difference in survival curves was analyzed by log-rank (Mantel-Cox) test (*P* = 0.002). **d** Mice treated with **CHD3**^**NEMO**^ for 24 h have attenuated NF-κB signaling in tumor cells.

Encouraged by the efficacy of **CHD3**^**NEMO**^ in cellular assays, we tested its anti-tumor activity in vivo. We injected BC-3-luc PEL cells into the peritoneal cavity of NOD-SCID mice and monitored tumor burden by bioluminescence imaging. After tumor engraftment, mice were randomized then treated with vehicle alone (*n* = 10) or with 20 mg/kg of **CHD3**^**NEMO**^ (*n* = 5) for 9 consecutive days. All mice in the vehicle arm showed clear tumor growth, however; mice in the treated group showed a remarkable delay in tumor growth without any observed toxicity (Figs. 6a and b, *p* < 0.05 at day 20). Moreover, Kaplan-Meier analysis showed that mice treated with **CHD3**^**NEMO**^ had a significant survival advantage compared to the control group (Fig. 6c, *p* < 0.05). To assess NF-κB signaling in vivo, we treated mice that had developed PEL with **CHD3**^**NEMO**^ for 24 h, and recovered tumor cells. We found significantly reduced phospho-IKKα/***β***, phospho-p65, phospho-IκBα and a decrease in vFLIP and NEMO levels in **CHD3**^**NEMO**^ treated mice, thus demonstrating that vFLIP inhibition in vivo leads to modulation of NF-κB signaling (Fig. 6d). Although the overall performance of our peptide is highly encouraging, further optimization of delivery and dosing will be essential to achieve more robust responses in vivo.

## Discussion

The topological complexity of NEMO-mediated protein-protein interactions underscores the difficulty in identifying small molecule leads or short peptides. The NEMO protein is characterized by a large and flat binding surface containing dispersed critical binding residues over a long coiled coil architecture^49^. We rationally designed a potent NEMO coiled coil mimetic, **CHD3**^**NEMO**^, to bind the viral oncoprotein vFLIP and disrupt its role in the pathogenesis of PEL as a single agent. Our studies demonstrate that a coiled coil mimic which captures the complex binding epitope of scaffolding proteins is required for potent inhibition^50^. Engineering of coiled coil epitopes required a new crosslinking strategy for the stabilization of the desired conformation^36,37^. Stability of coiled coils is intimately tied to the length of the individual peptide strands^37^. Our synthetic strategy to generate crosslinked helix dimers or CHDs affords minimal mimics of helical tertiary structures that are stable to proteases and enter live cells. Our approach offers one of a handful of approaches that provide conformationally and proteolytically stable coiled coil mimics^6,51^. To our knowledge, this is the first example of a helical tertiary structure mimic that targets an intracellular protein-protein interaction in cellular and animal models. Extensive prior efforts with engineered coiled coil mimics have largely been restricted to extracellular protein targeting^52,53^.

We found that a coiled coil mimic was required for vFLIP-NEMO disruption because our attempts to identify small molecules that inhibit the target complex only led to non-specific ligands. Furthermore, a constrained helix which mimics one of the two NEMO coiled coil strands also failed to yield a potent inhibitor, highlighting the need for ligands that engage surfaces not accessible to small molecules or single helix mimics. Our results with the helix constrained by a main-chain hydrogen bond surrogate^35^, are consistent with earlier reports of a NEMO peptide that was similarly constrained into an α-helical conformation using the side chain stapling method in that neither was very effective, albeit different experimental systems were used^19^. The optimal coiled coil mimic, **CHD3**^**NEMO**^, required computational optimization and design of non-natural contacts to engage vFLIP. We utilized a battery of biophysical and biochemical assays to characterize the mimic and its potential to bind vFLIP. We also demonstrated the compound’s efficacy in cellular assays and in a xenograft mouse model.

**CHD3**^**NEMO**^ inhibited vFLIP-mediated NF- κB transcriptional activity and disrupted the NEMO-vFLIP complex in competitive pull-down assays. We also show that the binding between these two proteins is essential for preventing their proteasomal-mediated degradation, and for the integrity of the NF-κB signalosome complex. The intimate role of ubiquitination and proteasomal degradation as important regulatory mechanisms in NF-κB signaling has been critically explored^54^. The designed mimic allows direct observation of the role of a viral oncoprotein in hijacking an important IKK mediator to perpetuate NF-κB signaling and protect against its proteasomal degradation.

**Fig. 7:**
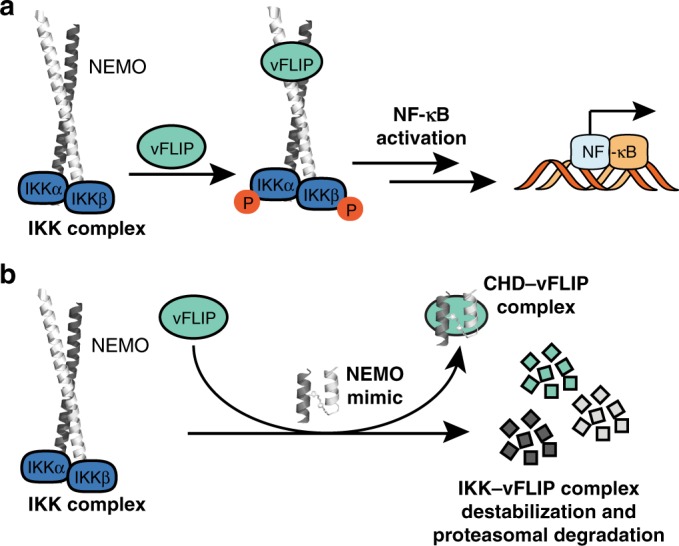
Model for CHD3^NEMO^ mechanism of action. **a** vFLIP activates NF-κB by engaging NEMO. **b** **CHD3**^**NEMO**^ (NEMO mimic) can directly bind vFLIP and compete with NEMO-vFLIP complex formation leading to destabilization of the IKK signalosome and inhibition of vFLIP-induced NF-κB activation in PEL cells.

Inhibition of the vFLIP–NEMO complex also decreased vFLIP stability. We anticipate that the reduction in vFLIP levels will impact other downstream interactions that vFLIP participates in to promote tumorigenesis. Figure 7 shows a model of **CHD3**^**NEMO**^ inhibition of vFLIP-induced NF-kB activation. A recent study by Choi et al.^47^, showed that inhibition of vFLIP’s interaction with the mitochondrial protein MAVS on the peroxisomes decreases vFLIP expression and leads to PEL cell death, and that MAVS is essential but not sufficient for vFLIP stabilization. **CHD3**^**NEMO**^ provides an important single-agent chemical tool for analyzing the broader roles of vFLIP and NEMO in promoting tumorigenesis and regulating the inflammatory response.

A synthetic compound that directly inhibits a central signaling node provides an alternative to genetic approaches for studying biological networks. A recent study has questioned the role of NEMO and other essential NF-κB subunits (P65 and P50) in primary effusion lymphomas^26^. However, we find that **CHD3**^**NEMO**^ is toxic to all PEL cell lines that we tested including BC-1 and BCBL-1 which Manzano et al.^26^ found do not require NEMO for survival. This suggests that vFLIP degradation by **CHD3**^**NEMO**^ itself may lead to additional effects that go beyond NEMO inactivation, and may include other proteins in the signaling complex, including those in the NF-κB alternative pathway^28^. Our data provides molecular insights into the various mechanisms that regulate vFLIP protein and its ability to activate NF-κB and indicate that vFLIP stability is complex-dependent. The mechanistic data provide new insights into how other viral oncoproteins that bind NEMO, like Tax and vCLAP encoded by human T-cell leukemia virus type 1 (HTLV-1) and equine herpesvirus-2^2,55^, modulate the IKK signalosome to induce persistent NF-kB activation.

**CHD3**^**NEMO**^ induced apoptosis supporting the premise that NEMO-vFLIP complex inhibition is critical to regulating NF-κB activity and its target genes. Importantly, the activity of the designed ligand is sequence selective as the closely related crosslinked peptides, **CHD2**^**NEMO**^ and **CHD4**^**NEMO**^ had a diminished ability to compete the complex, inhibit NF-κB activation and induce cytotoxicity, underscoring the specificity imparted by the designed hot spot residues. Coiled coil-mediated protein interactions often display complex epitopes suggesting that the CHD strategy may prove useful in targeting this intractable class of interactions^50^.

**CHD3**^**NEMO**^ significantly reduced tumor volume of a PEL xenograft model and granted survival advantage over the untreated group without any observed toxicity. **CHD3**^**NEMO**^ treated mice also had attenuated NF-κB signaling demonstrated by the significant downregulation of signaling components. This supports our hypothesis that the complex binding epitope of coiled coils can be mimicked using CHDs, and targeting the NEMO-vFLIP interaction and modulating IKK signalosome stability is a promising approach for treating KSHV^+^-associated malignancies, including KS, that also express oncogenic vFLIP^19,30,31^.

## Methods

### Peptide synthesis

Peptides were synthesized on a GYROS Protein Technologies Prelude X instrument using standard Fmoc solid phase chemistry with Knorr Amide MBHA resin. Peptides were cleaved from the resin using 95% trifluoroacetic acid, 2.5% TIPS, and 2.5% H_2_O, and purified by reversed-phase HPLC (gradient 15–60 acetonitrile/water with 0.1% TFA over 60 min) and characterized by MALDI-TOF. The compound characterization data is included in Supplementary Information (Supplementary Table 6 and Supplementary Fig. 12).

### Fluorescence polarization assay

The relative affinity of **FITC-CHD3**^**NEMO**^ to MBP-vFLIP were determined using fluorescence polarization-based direct binding assay. The polarization experiments were performed using a DTX 880 Multimode Detector (Beckman) at 25 °C with excitation and emission wavelengths of 485 and 525 nm, respectively. Each polarization experiment was performed in a 96-well round bottom plate (Greiner) in assay buffer: 20 mM Tris-HCl, 150 mM NaCl, 0.5 mM TCEP, 10%  glycerol, 0.1% pluronic acid, pH 8. The binding affinity (*K*_D_) values reported for each peptide are from experiments performed in triplicate. Raw values were fit to a sigmoidal dose-response nonlinear regression model in Graphad Prism 6.0.

All binding experiments with FITC-labeled CHD peptide to MBP-vFLIP were performed under the same conditions. Briefly, serial dilutions of MBP-vFLIP were made from 75 μM into 100 nM of FITC-labeled CHD peptide in assay buffer. *K*_D_ was determined using the following equation^56^:

$${\mathrm{K}}_{\mathrm{D}}=\left({{\mathrm{R}}_{\mathrm{T}}\times\left({1-{\mathrm{F}}_{\mathrm{SB}}}\right)+{\mathrm{L}}_{\mathrm{ST}} \times {\mathrm{F}}_{\mathrm{SB}}^{2}}\right)/\left.{\mathrm{F}}_{\mathrm{SB}}\right) - {\mathrm{L}}_{\mathrm{ST}}$$

where,

*R*_T_ = Total concentration of MBP-vFLIP

*L*_ST_ = Total concentration of FITC-CHD peptide

*F*_SB_ = Fraction of bound FITC-CHD peptide

Statistics are represented at 95% confidence level.

### Circular dichroism spectroscopy

CD spectra were recorded on a Jasco J-1500 Circular Dichroism Spectrophotometer equipped with a temperature controller using 1 mm length cells and a scan speed of 4 nm/min at 298 K. The spectra were averaged over 4 scans with the background subtracted to the analogous experimental conditions. Each sample was prepared in a 50 mM potassium fluoride solution in water (pH 7.4) to a final concentration of 20 μM. The concentrations of each peptide were determined by the UV absorption at 280 nm.

### Serum stability assay

Proteolytic stability of **CHD3**^**NEMO**^ was determined using 25% fetal bovine serum in RPMI. Time points of 30 min, 1 h, 2 h, 8 h, and 24 h were analyzed in triplicate. Each reaction was started upon addition of FBS to 60 μM peptide. Reactions were quenched at time points with addition of 100% EtOH, chilled on ice for 10 min, and pelleted at 11,752 × *g*. Supernatant was injected onto an Agilent analytical HPLC equipped with an XTerra RP18 3.5 µm 2.1 × 150 mm column (Part No. 186000410) and visualized at 220 nm. Integration of peak areas was used to determine percent degradation of peptides. Mass of cleaved products were determined using Matrix assisted laser desorption ion time of flight (MALDI-TOF) spectroscopy.

### Cell lines and culture conditions

BC-1 and BC-3 PEL cell lines were established in the Cesarman laboratory from lymphomatous effusions as described previously^13,57^. BCBL-1 was obtained from the AIDS and Cancer Specimen Bank. Namalwa Burkitt lymphoma and Hela cell lines were purchased from American Type Culture Collection (ATCC 70016358). All cell lines were tested and found to be free of mycoplasma. Cells were grown in RPMI 1640 (GE Healthcare) supplemented with 10% (Namalwa) or 20% heat-inactivated FBS (Atlanta Biologicals) and 50 µg/mL of gentamicin (Atlanta Biologicals). vFLIP WT and vFLIP NF-kB dead mutant inducible cell lines were established by cloning WT FLAG-tagged vFLIP and mutant vFLIP into cloned pLVX-Tetone-puro vector backbone which is a component of the XLenti-X™ Tet-One™ Inducible Expression system (Clontech). Mutant vFLIP contains three amino acids mutation at position 57 where 3 amino acids ECL are replaced with three alanines, AAA. These plasmids were packaged in 293T cells and lentiviral particles were used to transduce parental Namalwa cell line. Stable transduced cell lines were established by puromycin selection at 1 µg/mL. Induction of WT vFLIP or mutant vFLIP expression was attained by adding doxycycline (Sigma) at 1 µg/mL.

The double reporter cell line BC3NFRen-luc#3 was generated by transduction of this cell line using a lentiviral construct expressing renilla luciferase controlled by a constitutive promoter (retroviral LTR). These cells were maintained in RPMI-1640 supplemented with 20% FBS and 50 μg/mL Gentamicin, as well as 1.2 mg/mL Geneticin or G418 (Life Technologies) to maintain clonal selection.

For HeLa TNF α stimulation experiment, cells were pretreated with increasing doses of **CHD3**^**NEMO**^ (0, 5, 25, and 40 μM) in the absence of serum and were supplemented with serum after 3 h and peptide treatment continued for a total of 24 h. The next day, cells were treated with PBS or stimulated with 20 ng/mL of TNFα (PeproTech) for 30 min followed by cell lysis, protein extraction and immunoblotting for NF-κB signaling components.

### Protein expression and purification

vFLIP (1-178) and NEMO (150-272) were cloned into pET28a vector (kindly provided by Dr. Hao, Children’s Boston Hospital). his-NEMO pET28a (150-272) and pET28a his-MBP vFlip (1-178) were transformed into BL21(DE3) cells (Invitrogen) and plated on kanamycin plates. Colonies were picked and cultured in LB broth containing kanamycin (50 μg/mL). Cultures were inoculated to 1liter of LB broth with kanamycin and incubated at 37 °C for 3 h then allowed to cool down at RT for 1 h. Cultures were placed in incubator at 18 °C and induced with 0.1 mM IPTG where optical density was 0.5 and incubated overnight with shaking. Next day, cells were pelleted and resuspended in lysis buffer and lysed using microfluidizer in the presence of PMSF. Supernatants were spun down using ultracentrifuge with vaccum at 22,600 × *g* for 50 min (TI-45 rotor). Proteins were purified on a pre-equilibrated nickel column Ni-NTA Super Flow resin (Qiagen) and eluted using elution buffer (20 mMTris-HCl, 250 mM imidazole, 150 mM NaCl, 0.5 mM TCEP). Further purification of the protein was performed using gel filtration column (superdex200) and eluted in size exclusion buffer (20 mM Tris-HCl pH = 8, 150 mM NaCl, 10% glycerol and 0.5 mM TCEP). HPLC fractions were analyzed on 15% SDS-PAGE gels. Proteins were concentrated (calculated assuming an extinction coefficient), aliquoted and flash frozen at −80 °C. His-NEMO protein was biotinylated using EZ-Link iodoacetyl-PEG_2_-Biotin kit (cat. 21334, Thermoscientific) following manufacturer’s instructions and biotinylated protein was purified on a zeba spin desalting column (cat. 89891, Thermoscientific). Biotin incorporation was quantified using the HABA (4-hydroxyazobenzene-2-carboxylic acid (cat. 28005, Pierce Biotin Quantitation kit) to yield 1.64 moles of biotin/mole of protein.

### TR-FRET competition assay

For the competition assay, two-fold serial dilution of the different NEMO mimetics were prepared in DMSO then diluted in TR-FRET buffer to 5× and added in triplicates to a 384 low volume well plate. His-MBP vFLIP diluted to 5× in TR-FRET buffer (250 nM) was then added to each well and incubated for 15 min at RT. His-Biotinylated NEMO diluted to 5× in TR-FRET buffer (250 nM) was the added to the mixture followed by addition of 5× or 200 nM) streptavidin-XL665 and (5× or 1 nM) of the antibody-tagged fluorophore anti-MBPK labeled with Europium cryptate). The final concentration of the NEMO mimetics ranged from 0.195 μM to 100 μM. Total assay volume was 20 μL. The plate was incubated for 1 h at RT then read using BioTek Synergy NEO. Titration of non-biotinylated NEMO (0.0195-10 μM) was used as a positive control in every run. The effect of the peptides on vFLIP/NEMO interaction was normalized to the control and expressed as percent inhibition (% of control):

$${\hbox{\%}} {\;} {\mathrm{of}} {\;} {\mathrm{control}} = {\mathrm{FRET}}_{\mathrm{CHD}}-{\mathrm{FRET}} _{\mathrm{background}}/{\mathrm{FRET}}_{\mathrm{control}} - {\mathrm{FRET}}_{\mathrm{background}}$$

where FRET _control_ is the TR-FRET signal in DMSO treated wells (highest signal) and FRET _background_ is TR-FRET signal in wells containing the highest concentration of non-biotinylated NEMO (10 μM) which provides the lowest signal. Normalized TR-FRET data was plotted using Graphpad Prism.

### Cell viability assays

Cell viability assays were performed by plating log-phase BC-1, BC-3 and BCBL-1 PEL cells or Namalwa Burkitt lymphoma cell line in RPMI complete media in serum free medium at a density of 1 × 10^5^ cells/mL after which cells were treated with DMSO or a range of concentrations of NEMO mimetics varying from from 5 nM to 50 μM. Media was supplemented with 20% FBS 3 h post-peptide treatment. ATP content which correlates with metabolically active cells was measured using CellTiter-Glo kit (Promega, Madison, WI) at 24 and 72 h post-treatment. The LC_50_ for each NEMO mimetic in each cell line was determined using GraphPad Prism.

### NF-κB reporter assay

Exponentially growing BC-3-derived reporter cell lines (BC3-NFκB-luc#3), were resuspended in RPMI-1640 complete media plus 1.2 mg/ml selection antibiotic G418 in the absence of any serum and plated in a 96-well tissue culture microplate at 1 × 10^5^ cells/mL. Cells were then treated with DMSO or varying concentrations of **CHD2**^**NEMO**^, **CHD3**^**NEMO**^ or **CHD4**^**NEMO**^ peptides (at final concentration of 1 μM 5 μM, 10 μM or 25 μM). As a positive control, BC3NFκB-luc#6 cells were treated with DMSO or 1 μM or 10 μM of the HSP90 inhibitor PU-H71 or 5 μM and 10 μM of BAY-11in the presence of serum. 3 h post-treatment with the different peptides, 10% FBS was added to the media. The luciferase activity was measured 5 h and 24 h post-treatment using Dual-Glo Luciferase assay system (Promega, Madison, WI), according to the manufacturer’s instructions.

### Co-immunoprecipiation

FLAG-tagged WT vFLIP or a vFLIP NF-κB dead mutant (vFLIP^AAA(58-60)^) inducible Namalwa Burkitt lymphoma cell lines were used. Expression of WT vFLIP or mutant vFLIP was attained by treating cells with 1 µg/mL doxcycline for 24 h. Next day, cells expressing WT vFLIP were seeded in serum-free media and treated with DMSO or increasing concentrations of **CHD2**^**NEMO**^, **CHD3**^**NEMO**^ or **CHD4**^**NEMO**^ NEMO peptides (at a final concentration 5 μM, 25 μM or 50 μM) in the presence of 1 µg/mL doxcycline to enable continuous expression of vFLIP. 4 h post-treatment, media was supplemented with 20%FBS and treatment continued for another 24 h. Next day, uninduced Namalwa WT vFLIP cell line, treated Namalwa WT vFLIP expressing cell lines, as well as Namalwa expressing mutant vFLIP and parental Namalwa cell lines were harvested, washed in PBS and lysed on ice for 30 min using CelLytic M lysis reagent (Sigma, cat. C2978) supplemented with protease inhibitor cocktail (calbiochem, cat 539134). Cells were spun down and some of supernatant was saved for input and the rest was immunoprecipitated overnight using anti-FLAG M2beads (Sigma, cat. A2220) that were pre-equilibrated with the same buffer. Next day, beads were washed with CelLytic buffer five times and protein complexes were eluted using SDS lammeli buffer and boiling at 95 °C. For the pull down in the presence of MG-132, WT vFLIP cell lines were treated with DMSO or 5, 10, or 25 μM of **CHD3**^**NEMO**^ in the presence or absence of 0.5 μM of the proteasomal inhibitor MG-132 for 24 h. Cells were lysed the next day and protein concentration was quantified using BSA assay and equal amounts of protein were used for immunoprecipitation using anti-FLAG M2beads.

For the BC-1 immunoprecipitation study, cells were treated with DMSO, 5, 25, 50 μM of **CHD3**^**NEMO**^ in serum-free media and supplemented with 20% FBS 4 h post-initiation of treatment. 48 h post-treatment, cells were lysed in CelLytic M lysis reagent and incubated with protein A/G beads (sc-2003) and immunoprecipitated using IgG isotype control (genetex GTX35035) or NEMO antibody (Santa Cruz sc-8330). Next day, beads were washed with CelLytic buffer five times and protein complexes were eluted using SDS lammeli buffer and boiling at 95 °C.

### Immunoblotting

Cells were lysed in RIPA buffer (Pierce) in the presence of protease cocktail inhibitor (calbiochem) and halt phosphatase inhibitor (Thermo scientific). Protein lysates were quantified using Pierce BCA assay (Thermo scientific) and separated using pre-casted 10% sodium dodecyl sulfate-polyacrylamide gel electrophoresis SDS-PAGE gel (Bio-rad). Proteins were transferred to a PVDF membrane and blocked in 5% w/v nonfat dry milk-TBST for 1 h at room temperature. Membrane was then washed and incubated overnight with primary antibodies diluted in 5% BSA-TBST overnight.

### Antibodies

The following primary antibodies were used: NEMO (1:500 or 1:1000, GTX107582, lot #39946), GAPDH (1:25,000, GTX100118, lot #41577) from Genetex. phospho IKKα/β (Ser176/180) (1: 500, clone 16A6, cat #2697 S, lot #13), phospho p65 (Ser536) (1:500, clone 93H1, cat #3033 S, lot #6), total p65 (1:500, clone C22B4, cat #4764 S, lot #8), phospho IkBα (S32/36) (1:500, cat #9246 S, lot #23), IkB (1:800, cat #4814, lot #4), IKKβ (1:700, clone D30C6, cat #8943 s, lot #4) and HSP90 antibody (1:1000, 4877 T) from cell signaling technology. IKKα (1:300, H-744, sc-7218, lot #2903), NEMO (1:200, cat #sc-8330, lot #D0414) for pull down experiment, phospho p65 (1:200, sc-136548) for cell fractionation experiment and PCNA (1:500, cat # sc-56, lot #E2814) were purchased from Santa Cruz Biotechnology. Anti-Flag (1:1000, cat #600-401-383, lot #28976) from Rockland. A rat monoclonal antibody to vFLIP (1:200, clone 4C1) was kindly provided by Elisabeth Kremmer at Helmholtz Zentrum Munchen, Germany. Secondary anti-HRP rabbit antibody (1:5000, NA9340V, GE healthcare), Secondary anti-HRP mouse antibody (1:2000, NA931V, GE healthcare) and goat anti-rat IgG (H&L) HRP antibody (1:5000, 31470, ThermoFisher Scientific). Rabbit Trueblot anti-Rabbit IgG HRP (1:1000, Rockland 18-8816-3, lot 30172) to enable detection of NEMO in the immunoprecipitated BC-1 cells without interference of the heavy or light chains. Chemiluminescent signal was detected using enhanced chemiluminescence (ECL) substrate (Thermo Fisher Scientific) or Lumigen ECL Ultra followed by autoradiography

### Annexin V staining

BC-1 cells were treated with DMSO or increasing concentrations of the **CHD2**^**NEMO**^, **CHD3**^**NEMO**^, or **CHD4**^**NEMO**^ peptide for 1 h in the absence of serum then 20% FBS was added 1 h after serum starvation. Cells were harvested at 24 h or 48 h post-treatment, washed once in PBS and resuspended in Annexin V staining buffer (BD Pharmingen Catalog No. 556454) containing 3 μL/test AnnexinV-Alexa Fluor 647 (Thermo Fisher A23204) and 1 uL/test DAPI (Sigma D9542) and incubated at room temperature for 15 min in the dark. Data were acquired with a BD LSRII analytical flow cytometer and analyzed using FlowJo software. Necrotic/late apoptotic cells were defined as Annexin V^−^/DAPI^+^, AnnexinV^+^/DAPI^+^ and early apoptotic cells were defined as Annexin V^+^/DAPI^−^.

### PEL in vivo xenograft mouse model

10 × 10^67^ BC3NFRen-luc#3 were injected intraperitoneally into 4-6 week-old male NOD/SCID mice (Jackson Laboratory, stock 001303). Mice were followed by in vivo *luciferase* imaging using IVIS Imaging system (PerkinElmer) to confirm tumor engraftment after which mice were randomized to vehicle (*n* = 12) and **CHD3**^**NEMO**^ treated groups (*n* = 5) with average tumor burden distributed evenly across the groups. Mice were treated intraperitoneally with vehicle (PBS–0.05%Tween-80) or with the **CHD3**^**NEMO**^ peptide (20 mg/kg/day) for 9 consecutive days. We monitored tumor burden or bioluminescence (photons/s/cm^2^/steradian) by live imaging and weighing, with the sacrificial endpoint determined to be a net gain or loss of 10% body weight over a week. We assessed the effect of **CHD3**^**NEMO**^ on overall survival by Kaplan-Meier curves generated using GraphPad Prism software, and determined *p* values by analysis using log-rank (Mantel-Cox) tests.

### Study approval

All animal studies were conducted according to the IACUC protocols approved by Weill Cornell Medicine.

### Live cell confocal microscopy and analysis

BC-1 PEL cells in the exponential phase were resuspended in RPMI 1640 media supplemented with 50 μg/mL gentamicin in the absence of serum and treated with a final concentration of 0.125% DMSO or 500 nM FITC-labeled **CHD3**^**NEMO**^ at 37 °C. To test for mechanism of cellular uptake, **CHD3**^**NEMO**^ treatment was performed in cold temperature (4 °C) or in the presence of 10 mM sodium azide. Cells were then added to 35 mm glass bottom MatTek poly-lysine coated plates (p356c-0-10C) and immunofluorescence images were captured using LSM880 confocal miscroscope with Airyscan resolution detector, spectral detector and incubation. Images were processed using Fiji software (Supplementary Fig. 11).

### Design of NEMO mimics by AlphaSpace

AlphaSpace is a computational approach used to map the interface into a set of fragment-centric pockets^40^. Pockets are represented as geometric “alpha clusters”, which serve as 3-dimensional representations of the pocket and can be utilized to guide the selection or design of natural or non-natural residues to enhance pocket occupancy. This approach has been demonstrated previously in the optimization of a peptide inhibitor against a challenging protein-protein interaction target^41^.

Starting from the vFLIP-NEMO crystal complex structure (PDB Code 3CL3)^12^, we selected vFLIP (chain A) as the target interface and NEMO (chains D and E) as the template coiled coil to be optimized. Using AlphaSpace we mapped the surface of vFLIP to detect fragment-centric pockets at the NEMO interface and then characterized residue-centric pockets by associating alpha-atoms with their nearest residue in NEMO (Supplementary Fig. 11). This yields 6 interface pockets: 3 high-volume pockets (associated with Glu240 on *Helix 1*, Phe238 on *Helix 2*, and Lys246 on *Helix 2*), 2 moderate-volume pockets (associated with Ala233 on *Helix 1* and Asp242 on *Helix 2*), and 1 low-volume pocket (associated with His235 on *Helix 2*). All pockets and their associated residues are illustrated in Supplementary Fig. 11. The alpha-space volume and pocket occupancy data are listed in Supplementary Table 5. AlphaSpace calculations are performed with the software AlphaSpace1.0 (http://www.nyu.edu/projects/yzhang/AlphaSpace/). Suggested mutations to optimize the interface were selected or manually designed and evaluated using AlphaSpace.*Helix 2* is the primary binding helix in the native vFLIP-NEMO interaction. His235 and Phe238 both exhibit high pocket occupancy with *Pocket 6* and *Pocket 2,* respectively. Asp242 only partially occupies *Pocket 4* but is well-positioned to form dual-hydrogen bonds to pocket-lining residues in vFLIP: His82 and Tyr90. Lys246, however, is not observed to engage in a polar interaction with vFLIP, nor does it extend into the adjacent pocket. We proposed a mutation to arginine could reinforce the hydrogen bonding network of Asp242 by forming an intrahelical salt bridge that is well-accommodated by the crystal complex, by increasing pocket occupancy, and by promoting pi-cation stabilization with Tyr90 in vFLIP.

We detected a large volume of non-polar pocket space adjacent to *Helix 1* that is unoccupied in the native vFLIP-NEMO crystal complex. The moderate-volume *Pocket 5* can be targeted directly by tryptophan in a high-probability rotamer state by mutating Ala233. The high-volume *Pocket 1* adjacent to Glu240, however, is located beyond the reach of any natural amino acid. We designed a non-natural cyclohexyl amine as a glutamine derivative (Q^cy^) to both preserve the hydrogen-bond observed between Glu240 and the backbone of pocket-lining residue Phe53 in vFLIP and to extend the cyclohexyl group into the hydrophobic vFLIP pocket with good complementarity. All three suggested mutations are integrated into the **CHD3**^**NEMO**^ coiled coil mimic.

### Synthesis of hydrogen-bond surrogate NEMO mimic (HBS^NEMO^)

**HBS**^**NEMO**^ was synthesized as previously described^58^. Peptide sequences up to the i + 3rd residue of the parent strand were synthesized on solid phase on a GYROS Protein Technologies Prelude X instrument. A solution containing premixed o-nitrobenzesulfonyl chloride (10 eq) and 2,4,6-collidine (10 eq) in DCM was added to resin containing Fmoc-deprotected peptide. Resin was washed sequentially with dichloromethane, dimethylformamide and diethyl ether (3 × 5 mL each). Resin was dried overnight under vacuum. Dried resin, PPh_3_, and Pd_2_(dba)_3_ were flushed under inert argon for 30 min. The resin with reactants was swelled in THF, and allylmethylcarbonate was added to the reaction vessel. The solution was agitated at room temperature for 3 to 5 h under argon to afford allylated peptide. Resin was filtered and washed with DCM, DMF, 0.2 M sodium diethylcarbamate trihydrate in NMP, and diethyl ether (3 × 5 mL). The nosyl protecting group was then removed by the addition of 1,8-diazabicyclo[5.4.0]undec-7-ene (DBU, 5eq) and 2-mercaptoethanol (10 eq.) in DMF. Resin was washed with DMF, DCM, and diethyl ether (3 × 5 mL) and treated with the desired Fmoc amino acid (20 eq.), DIC (20 eq.) and HOAt (10 eq.) in DMF. The reaction was allowed to agitate at room temperature for 12 to 16 h. Resin containing elongated peptide was washed, and coupled to the desired Fmoc amino acid residue (5 eq.) and 4-pentenoic acid (5 eq.) with HBTU (5 eq.) and DIEA (10 eq.) in DMF. Ring-closing metathesis of bis-olefin was performed with Hoveyda-Grubbs II catalyst (20 mol%) in 1,2-dichloroethane under microwave irradiation at 120 °C for 10 min as previously described^59,60^. The ring-closing reaction was monitored by MALDI-TOF. Peptides were cleaved from the resin using 95% trifluoroacetic acid, 2.5% TIPS, and 2.5% H_2_O, and purified by reversed-phase HPLC (gradient 15–60 acetonitrile/water with 0.1% TFA over 60 min) and characterized by MALDI-TOF.

### Synthesis of crosslinked helix dimer NEMO mimic (**CHD3**^**NEMO**^)

Crosslinked helix dimers were synthesized as previously described with minor modifications^36^. Parent peptide (0.25 mmol) Helix 1 was synthesized on a GYROS Protein Technologies Prelude X instrument using standard Fmoc solid phase chemistry with Knorr Amide MBHA resin. Fmoc-Glu(OAllyl)-OH was incorporated into precursor parent peptide, Helix 1. The resin bearing Helix 1 was transferred to a fritted polypropylene SPE tube and washed with DMF, DCM, and MeOH (3 × 5 mL). Allyl deprotection was performed using Pd(PPh_3_)_4_ (3 equiv) in a solution of chloroform: acetic acid: N-methylmorpholine (37:3:1). After 3 h, the resin was washed again with DCM, DMF, MeOH (3 × 5 mL each). Following addition of PyBOP (3 equiv) and DIPEA (3 equiv) for 10 min, cyclohexylamine (6 equiv) was added resulting in Q^Cy^-installed peptide. MALDI-TOF confirmed complete amidation of glutamate. The resin was washed, transferred to a microwave tube, and subsequently swelled in 3 mL of NMP and the bis-alkyne propargyl ether (257 μL, 2.5 mmol, 10 equiv) was added.

A solution of CuSO_4_ (20 mg, 0.125 mmol, 0.5 equiv) dissolved in 500 μL of water was separately prepared. To this solution, Tris[(1-benzyl-1H-1,2,3-triazol-4-yl)methyl]amine (132 mg, 0.25 mmol, 1 equiv) dissolved in 1 mL of NMP was added. This mixture was added to a solution of sodium ascorbate (495 mg, 2.5 mmol, 10 equiv) prepared in 1.5 mL of water. The resulting mixture was pipetted into the microwave tube containing propargyl ether and peptide. A magnetic stir bar was added, and the reaction mixture was subjected to microwave irradiation at 85 °C for 45 min, after which the resin was transferred to a fritted polypropylene SPE tube and washed with a 20 mM solution of sodium diethyldithiocarbamate in water (3 × 15 mL) followed by NMP (3 × 15 mL). A microcleavage of resin (95% trifluoroacetic acid, 2.5% TIPS, and 2.5% H_2_O) showed the starting material to be consumed after one reaction. Helix 2 was synthesized using the same protocol. Importantly, no copper catalyzed azide alkyne cycloaddition (CuAAC) was performed on Helix 2, leaving a functional azide handle. Each peptide was treated with a solution containing 95% trifluoroacetic acid, 2.5% TIPS, and 2.5% H_2_O. Separately, both peptides were precipitated with cold diethyl ether and dried under a stream of nitrogen gas. HPLC purification (gradient 15-65 acetonitrile/water with 0.1% TFA over 60 min) and lyophilization yielded peptide as a white powder characterized by MALDI-TOF. Unconstrained peptide yield, sequence dependently, 25 mg of peptide from a 0.25 mmol scale.

For reaction on 1 μmol scale, purified peptides were dissolved in a ratio of 1:2 by weight (azide:alkyne) with final concentration at least 200 μM in 1 mL NMP and diluted with 1× PBS pH 7.4 (1:4) to give 4 mL of reaction solvent. 10 μL of a 10× solution of CuSO_4_ (16 mg, 100 μmol, 100 equiv) dissolved in 1 mL of 1× PBS was prepared separately. To the CuSO_4_ solution, Tris[(1-benzyl-1H-1,2,3-triazol-4-yl)methyl]amine (TBTA) (2.8 mg, 5 μmol, 5 equiv) dissolved in 100 μL of NMP was added. Sodium ascorbate (10.2 mg, 50 μmol, 50 equiv.) was dissolved in 1 mL of 1× PBS. The TBTA-CuSO_4_ soltion was added to the peptide mixture, followed by Sodium ascorbate solution. The reaction was allowed to proceed for 4 h. Crude reaction mixture was filtered. HPLC purification (gradient 15–65 acetonitrile/water with 0.1% TFA over 60 min) and lyophiliziation yielded peptide as a white powder characterized by MALDI-TOF.

### Synthesis of fluoroscein isothiocyanate (FITC) labeled peptides

The FITC analogs of **CHD-2**^**NEMO**^ and **CHD-3**^**NEMO**^ were synthesized. Parent CHD monomers were synthesized as described above. Prior to acetyl capping, Fmoc-β-alanine was added to the N-terminus of Helix 1. Following deprotection with 20% piperidine in NMP and washing with DMF, DCM, and MeOH (3 × 5 mL), FITC (1.2 equiv) and DIEA (2 equiv) were added to the solid phase tube and gently agitated for 2 h. The reaction was washed with DMF, DCM, and MeOH (3 × 5 mL), and characterized by MALDI-TOF. CHD synthesis and characterization proceeded as described earlier^36,37^.

### Analytical size exclusion chromatography

Peptides and standards were prepared at 10 mg/mL in 2× PBS supplemented with 10% ACN. Samples were injected onto an Agilent analytical HPLC equipped with a Superdex 30 Increase 3.2/300 column (Part No. 29219758) and visualized at 220 nm. Flow rate 200 µL/min over 60 min using 2× PBS 10% ACN. The concentrations of each peptide were determined by the UV absorption at 280 nm.

### vFLIP protein half-life and stability

vFLIP protein stability and turnover was measured by treating Namalwa WT vFLIP and NF-kB dead vFLIP mutant cell lines in the presence or absence of 5 μM of the proteasomal inhibitor MG-132 (Calbiochem cat 474790) and 100 μg/mL of the protein synthesis inhibitor cycloheximide (Sigma-Aldrich, cat. 66-81-9). Cells were harvested at the indicated time intervals and lysed in RIPA buffer and supernatants were analyzed by immunoblotting.

### Cell fractionation

For the detection of nuclear and cytoplasmic fractions, 3 million BC-1 cells were washed in ice cold PBS and lysed in 200 μL of cold cell lysis buffer (buffer A: 20 mM HEPES [pH 7.9], 10 mM NaCl, 3 mM MgCl_2_, 0.5% Nonidet P-40, 10% glycerol, 0.2 mM EDTA, Protease Inhibitor Cocktail III (Calbiochem) and Halt phosphatase inhibitor (Thermo scientific). Lysates were incubated on ice for 20 min and nuclei were pelleted by centrifugation. Supernatants containing cytoplasmic proteins were collected and stored at −80 °C. Pellets were washed one more time in 300 μL buffer A, and repelleted after which 300 μL buffer B (20 mM HEPES [pH 7.9], 20% glycerol, 0.2 mM EDTA, Protease Inhibitor Cocktail and phosphatase inhibitor). Nuclei were resuspended in buffer C (20 mM HEPES [pH 7.9], 0.4 M NaCl, 20% glycerol, 0.2 mM EDTA, Protease Inhibitor Cocktail III) and incubated on ice for 60 min and vortexed every 10 min. Nuclear extracts were collected after centrifugation for 15 min. For the detection of upstream

NF-kB signaling components, cytoplasmic fractions were collected using Cell biolabs kit (AKR-172) following manufacturer’s instructions.

### Statistical methods

In all cases in which 2 groups were compared, unpaired two-tailed Student’s *t* tests or log-rank tests were used to determine significance. ANOVA test was used when comparing 3 groups. A *P* value less than 0.05 was considered statistically significant. All bar and line graphs show mean ± SEM or mean ± SD of 2 or 3 independent experiments as indicated in the figure legends. Graphpad Prism 6.0 was used for analysis.

### Reporting summary

Further information on research design is available in the Nature Research Reporting Summary linked to this article.

## Supplementary information

Supplementary information

Reporting Summary

## Data Availability

There are no restrictions on data availability, and all other relevant data are available from the corresponding author on request. Raw blots corresponding to each gel are included as Source Data.

## Source Data

Source Data
