# Supplementary material for: Modulation of virus-induced NF-κB signaling by NEMO coiled coil mimics
Source: Nat Commun. 2020 Apr 14;11:1786. doi: 10.1038/s41467-020-15576-3 (PMC7156456; doi:10.1038/s41467-020-15576-3)
Supplement: Supplementary file 1 — Supplementary information [file 41467_2020_15576_MOESM1_ESM.pdf]

Inhibition of Virus-Induced NF-κB Signaling by NEMO Coiled Coil Mimics

Jouliana Sadek,[1,6] Michael G. Wuo,[2,6] David Rooklin,[2] Arthur V. Hauenstein,[3] Seong Ho Hong,[2] Archana Gautam,[5] Hao Wu,[3] Yingkai Zhang,[2,4] Ethel Cesarman,[1*] and Paramjit S. Arora[2*]

[1]Department of Pathology and Laboratory Medicine, Weill Cornell Medical College, New York, NY 10065; [2]Department of Chemistry, New York University, New York, NY 10003; [3]Department of Biological Chemistry and Molecular Pharmacology, Harvard Medical School, Boston, MA 02115. [4]NYU-ECNU Center for Computational Chemistry, New York University−Shanghai, Shanghai 200122, China, [5]Icahn School of Medicine at Mount Sinai, New York, NY 10029-5674

[6]These authors contributed equally to this work. *e-mail: ecesarm@med.cornell.edu or arora@nyu.edu

Supplementary Information

Table of Contents

	Pages
Supplementary Figures and Tables	S2-S18
Supplementary References	S19

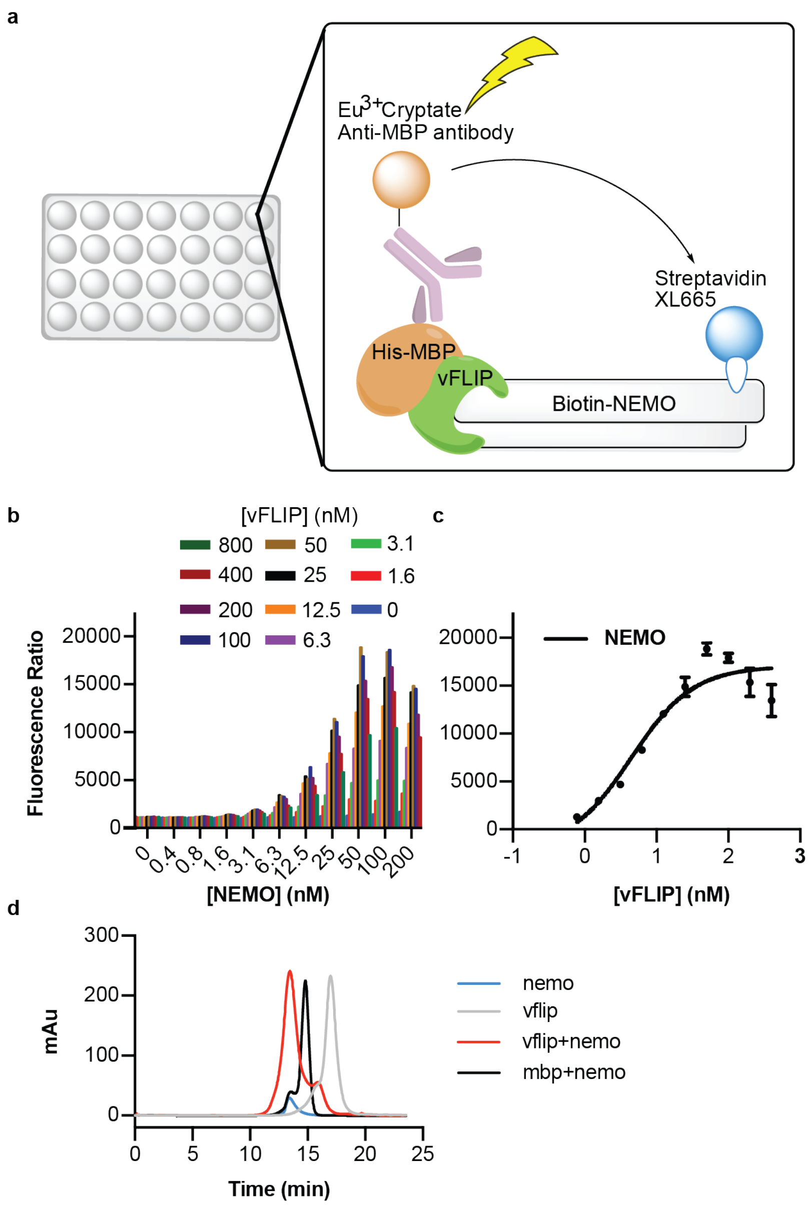

Supplementary Figure 1. Schematic representation of the Time-resolved Fluorescence Energy Transfer (TR-FRET) binding assay. (a) Complexes of recombinant his-MBP vFLIP, biotinylated His-NEMO, Europium cryptate conjugated anti-MBP antibody and strepavidin XL665 were used to generate proximity for a FRET signal between the donor fluorophore Europium cryptate and the acceptor fluorophore strepavidin XL665. The TR-FRET signal was calculated as a ratiometric measurement of the acceptor fluoroscence emission at 665nm over the donor emission at 620nm multiplied by 10000. (b) Matrix grid with serial dilutions of NEMO and vFLIP to optimize protein concentration and TR-FRET signal. Data plotted represent mean± SEM of two independent experiments (c) Direct binding assay of highest ratio signal (50 nM). (d) Size exclusion chromatography of NEMO-vFLIP complex indicating a shift to the left in absorbance at 280nm when NEMO binds specifically to vFLIP. MBP is used as a negative control.

Supplementary Table 1. vFLIP/NEMO high-throughput small molecule screening data summary.

Category	Parameter	Description
Assay	Type of assay	Time Resolved Fluorescence Assay (TR-FRET)
	Target	vFLIP/NEMO interaction
	Primary measurement	Fluorescence emission at 620nm for the donor (Eu^{3+} cryptate) and 665nm for the XL665 acceptor
	Key reagents	Purified his-MBP vFLIP, biotinylated 6xHis-NEMO, anti-MBP-Eu^{3+} cryptate (61MBPKAB, Cisbio) , Strepavidin XL665 (610SAXLB, Cisbio)
	Assay protocol	20 μl final volume in low-volume white 384-well plate (Greiner biointernational): biotinylated 6xHis-NEMO and 6xHis-MBP vFLIP proteins were diluted to a working concentration (5x or 250 nM) in TR-FRET buffer (20 mM Tris pH=7.5, 50 mM NaCl, 0.01% NP40, 0.5 mM TCEP, 0.1% BSA, 400 mM KF). The fluorophores anti-MBP-Eu^{3+} cryptate and Strepavidin XL665 were diluted to a working concentration of (5x or 5 nM) and (5x or 200 nM) respectively. Order of addition: 4 μl /well of 6xHis-MBP vFLIP was added to the screening plate containing 4 μl of compound diluted in buffer. 5 minutes later, 4 μl /well of biotinylated 6xHis-NEMO or 4 μl /well of buffer (negative control: vFLIP only) was added and the plate was incubated for 15 min at RT. 8 μl of the mixed diluted fluorophores anti-MBP-Eu^{3+} cryptate, and strepavidin XL665 were added to each well. The plate was spun down for 30 sec then incubated for 1 hr at RT. TR-FRET signal was detected using BioTek Synergy NEO.
	Additional comments	To determine the optimal signal, titrations of both 6xHis-biotinylated NEMO and 6xHis-MBP-vFLIP recombinant proteins and of the flurophores streptavidin-XL665 and the antibody-tagged fluorophore anti-MBPK labeled with Eu^{3+}cryptate (Cisbio) were carried out. Optimal incubation time and DMSO tolerance were also determined

Libraries screened	Library size<br><br>Library composition<br><br>Source<br><br><br><br><br><br>Additional comments	38,506 pure compounds<br><br>Low molecular weight screening compounds<br><br>LOPAC (Sigma, 1280 compounds), Enamine 3 (33,135 compounds), NIH clinical (727 compounds), HTRSC clinical (294 compounds), Prestwick (1109 compounds), Pharmakon (905 compounds). All compounds were dissolved in DMSO at 5 mM stock and stored at -28°C.<br><br>Library description www.rockefeller.edu/htsrc/libraries/
Screen	Format<br><br>Concentration tested<br><br>Plate controls<br><br><br><br>Compounds/Reagents dispensing system<br><br><br>Detection instrument<br><br>Assay Validation/QC<br><br><br><br><br>Normalization data	384-well plate<br><br>12.5 μM, 0.2% DMSO<br><br>6xHis-MBP vFLIP only (no NEMO, positive control), both 6xHis-MBP vFLIP and biotinylated 6xHis-NEMO (negative control)<br><br>Janus Automated Workstation with Nanohead (Perkin Elmer) for compounds; MultiDrop Combi with RapidStack (Thermo Scientific) for reagents<br><br>BioTek Synergy NEO multi-plate reader<br><br>Hit compounds were validated using dose-response titration (10 fold serial dilutions) in triplicates and a dose- response curve was fitted using CDD software. All confirmed hits were tested by HPLC-MS for purity and integrity<br><br>The TR-FRET signal or delta ratio was calculated as follows: Fluorescence of acceptor (665nm) / fluorescence of donor (620nm) *10000<br><br>Normalized percent inhibition (NPI) was calculated as the ratio of the sample to the positive control mean, after subtracting the background response, i.e. the negative control mean x 100 (NPI= (sample-mean of negative control)/ (mean of positive control- mean of negative control) x100. To evaluate the quality of the HTS TR-FRET assay, we calculated Z' factor which was determined to be 0.92. Z' was calculated according to the following formula:<br>Z' =1-[3*(standard deviation positive<br>control + standard deviation negative control)/(average positive control - average<br>negative control)]
Post-HTS analysis	Hit criteria<br><br>Hit rate<br><br>Additional assays<br><br>Confirmation of hit purity and integrity	Normalized percentage inhibition ≥ 30%<br><br>0.13%<br><br>Cytotoxicity assay using CellTiter-Glo<br><br>All confirmed hits were retested using HPLC-MS and found to be at least 85% pure. Powders were ordered and retested in dose-response curves

Supplementary Table 2. List of the top nine hits from the small molecule screen determined using the TR-FRET assay. Cytotoxic effect of the hits on cells that express vFLIP (BC-3 PEL cell line) compared to a non-vFLIP expressing cell line (IBL-1 immunoblastic lymphoma cell line) was examined. Data shown is representative of three replicate experiments.

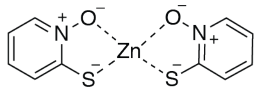

1

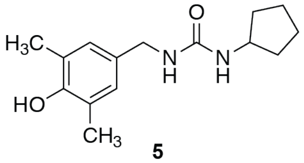

5

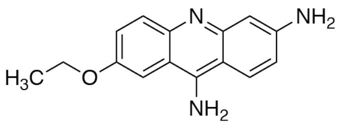

2

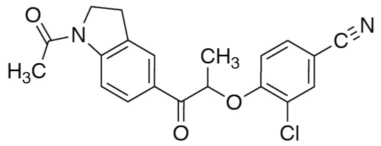

6

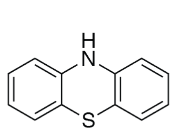

3

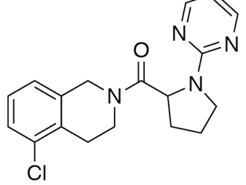

7

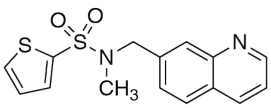

4

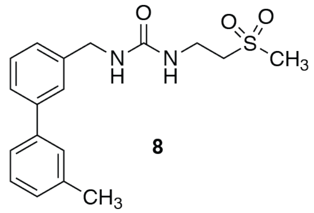

8

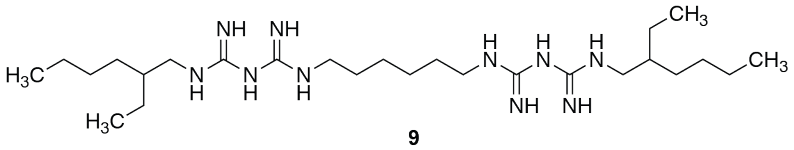

9

Compound	TR-FRET IC_{50} (μM)	BC-3 LC_{50} (μM)	IBL-1 LC_{50} (μM)
**1**	40.2	0.30	0.3
**2**	53.0	12.2	7.7
**3**	51.9	62.7	63.0
**4**	23.7	>100	>100
**5**	61.3	>100	>100
**6**	61.9	35.9	69.7
**7**	55.7	39.9	31.6
**8**	37.8	>100	>100
**9**	20.1	3.9	3.8

a

Compound	Sequence
UNC^{NEMO}	Ac-QVAYHQLFQEYDNHIK-NH_2
HBS^{NEMO}	XQVG*FQLFQEYDNHIK-NH_2

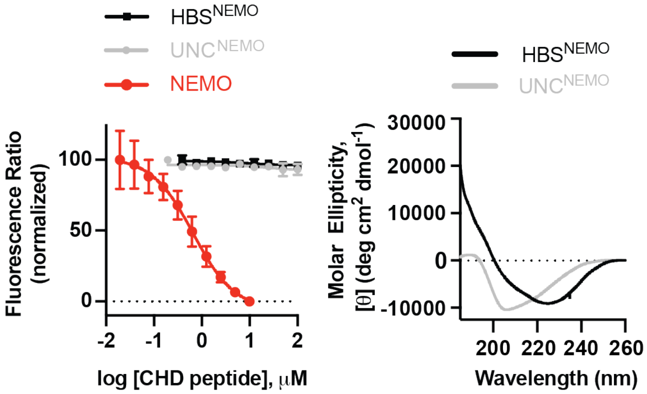

b

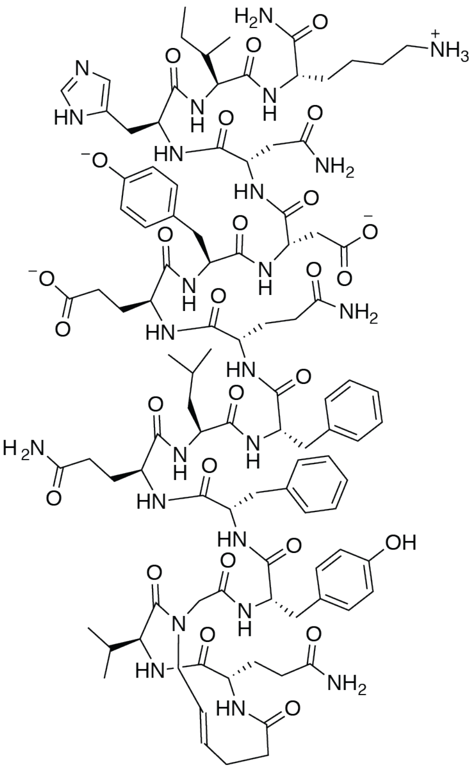

Supplementary Figure 2. (a) TR-FRET (left) and Circular Dichroism (CD) spectra (right) of **UNC^{NEMO}** and **HBS^{NEMO}.** Peptides used in CD studies were dissolved at 50 μM concentration in 50 mM Potassium Fluoride pH 7.4 supplemented with 10% TFE. TR-FRET data represent the mean± SD of (n=2) experiments each performed in triplicates. (b) Structure of **HBS^{NEMO}**.

Supplementary Table 3. Rosetta computational alanine scanning results for NEMO-vFLIP structure (PDB: 3CL3).[1] All hot spots were on Chain D from the PDB (Helix 2). Hot spot residues are highlighted in bold. Rosetta Energy Unit (R.E.U.) is approximately 1 kcal/mol.

Residue	ΔΔG (R.E.U.) from 3CL3
Q 231	0.94
V 232	-
A 233	-
**Y 234**	2.77
**H 235**	1.48
Q 236	-
L 237	-
**F 238**	3.75
**Q 239**	1.30
E 240	-
**Y 241**	1.18
D 242	0.28
N 243	-
H 244	-
**I 245**	1.03
K 246	0.68

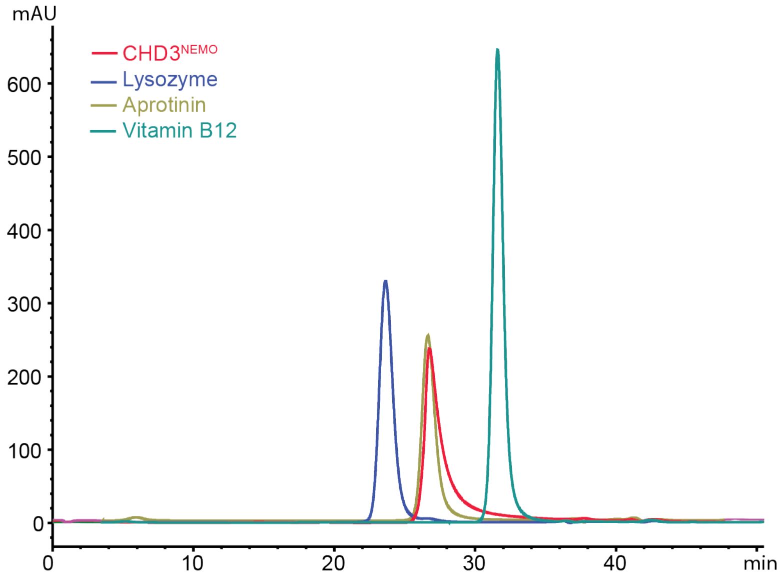

Supplementary Figure 3. Analytical size exclusion chromatography of **CHD3^{NEMO}**. Peptide standards lysozyme (14.3 kDa) and Aprotinin (6.5 kDa) and small molecule standards Vitamin B12 (1.4 kDa) are shown on the same plot. Solvent system: 2X PBS pH 7.4 10% Acetonitrile. Data is representative of two independent experiments (n=2).

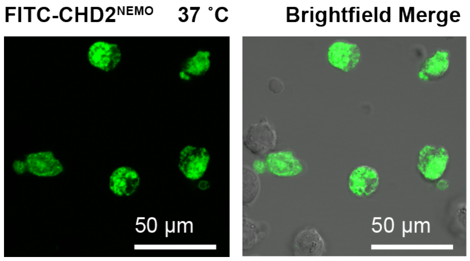

Supplementary Figure 4. Live confocal microscopy showing cellular uptake of FITC-labeled **CHD2^{NEMO}**. BC-1 PEL cells in the exponential phase were resuspended in RPMI 1640 media supplemented with 50 μg/ml gentamicin in the absence of serum and treated with a final concentration of 500 nM **FITC-CHD2^{NEMO}** at 37°C. Cells were then added to 35 mm glass bottom MatTek poly-lysine coated plates (p356c-0-10C) and immunofluorescence images were captured using LSM880 confocal microscope with Airyscan resolution detector, spectral detector and incubation. Images are representative of 2 independent experiments. Scale bar= 50 μm.

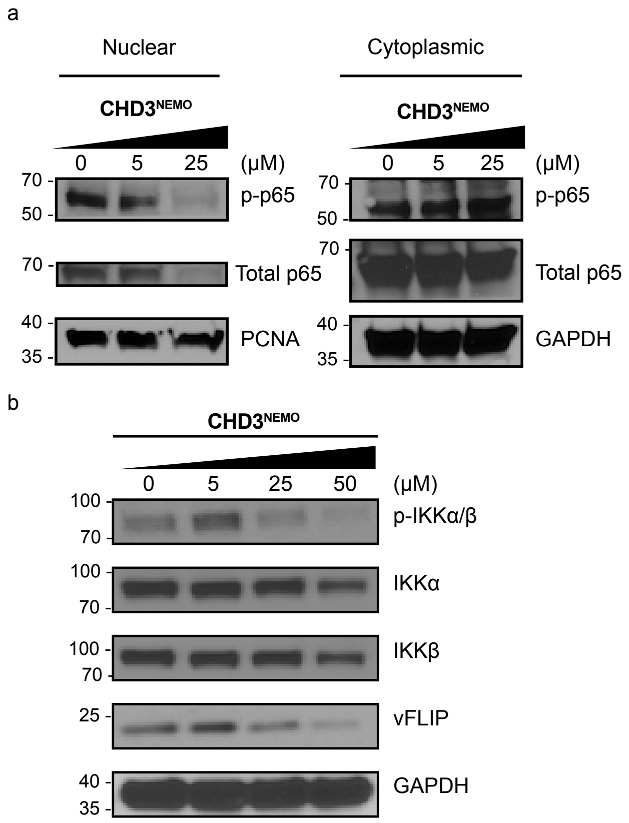

Supplementary Figure 5. CHD3^{NEMO} inhibits NF-κB signaling in BC1 PEL cell line. (a) Nuclear and cytoplasmic fractions from BC1 cell line showing that treatment with 25 μM of **CHD3^{NEMO}** for 24hrs inhibits p65 nuclear translocation. PCNA and GAPDH are used as loading controls. (b) Western blot analysis demonstrates **CHD^{3NEMO}** dose responsive modulation of NF-κB signaling components in BC1 cytoplasmic extracts. These are representative western blots of two independent experiments (n=2).

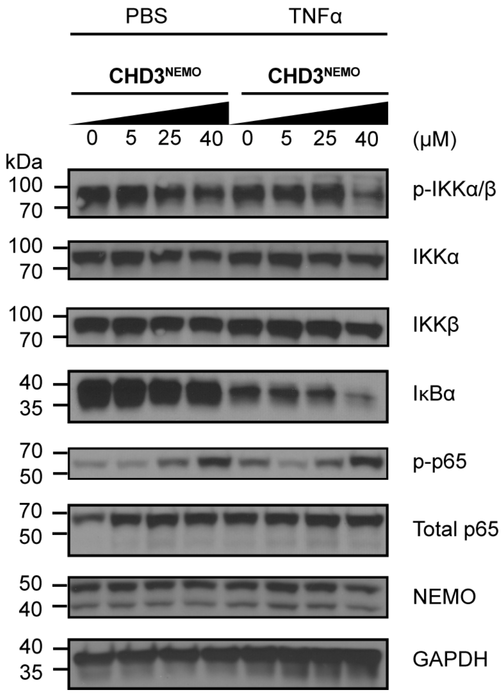

Supplementary Figure 6. CHD3^{NEMO} pretreatment does not block TNFα stimulation and NF-κB signaling in HeLa cells. Western blot analysis of total protein lysates of HeLa cells treated with increasing doses of **CHD3^{NEMO}** for 24hrs, then treated with PBS or TNFα 20ng/ml for 30min the next day followed by cell lysis and protein extraction. Western blots are representative of three independent experiments (n=3).

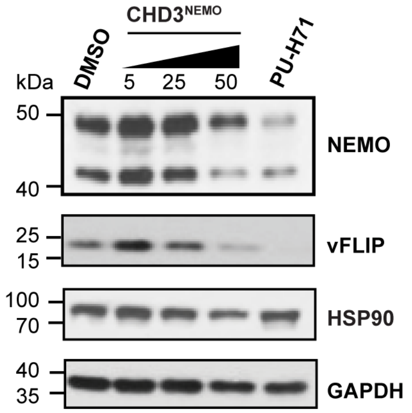

Supplementary Figure 7. **CHD3^{NEMO}** treatment downregulates vFLIP target expression in BC-1 PEL cells. Western blot using vFLIP rat 4C1 antibody showing that both vFLIP and NEMO protein levels decrease upon treatment with 50 μM **CHD3^{NEMO}**. The HSP90 inhibitor PU-H71 (2 μM) was used as a control since it destabilizes vFLIP and NEMO levels. GAPDH was used as a gel loading control. Data shown is a representative of two independent experiments (n=2).

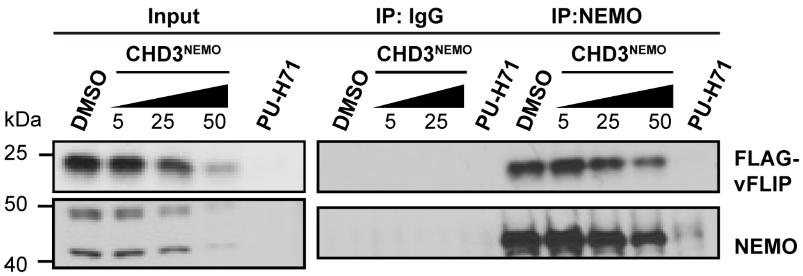

Supplementary Figure 8. CHD3^{NEMO} treatment decreases vFLIP association with NEMO in BC-1 PEL cells. Co-Immunoprecipitation assay with extracts of BC-1 cells treated with 0, 5, 25 and 50 μM of **CHD3^{NEMO}** for 48 hours and pulled down using NEMO antibody or IgG isotype control. PU-H71 (2 μM) was used a control. Data shown is a representative of two independent experiments (n=2).

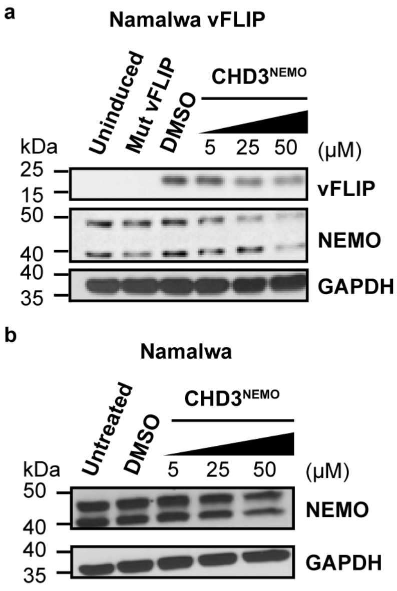

Supplementary Figure 9. Effect of CHD3^{NEMO} on NEMO levels in Namalwa cells expressing vFLIP target. Western blot using cell lysates from (a) vFLIP inducible Namalwa vFLIP and (b) Namalwa parental cell lines treated with DMSO or increasing concentrations of the CHD3^{NEMO} peptide. vFLIP and NEMO protein levels decrease in a dose-dependent manner in the vFLIP inducible Namalwa upon treatment with 25 and 50µM dose of the CHD3^{NEMO} peptide whereas NEMO levels decrease at higher concentrations. Data shown is a representative of two independent experiments (n=2).

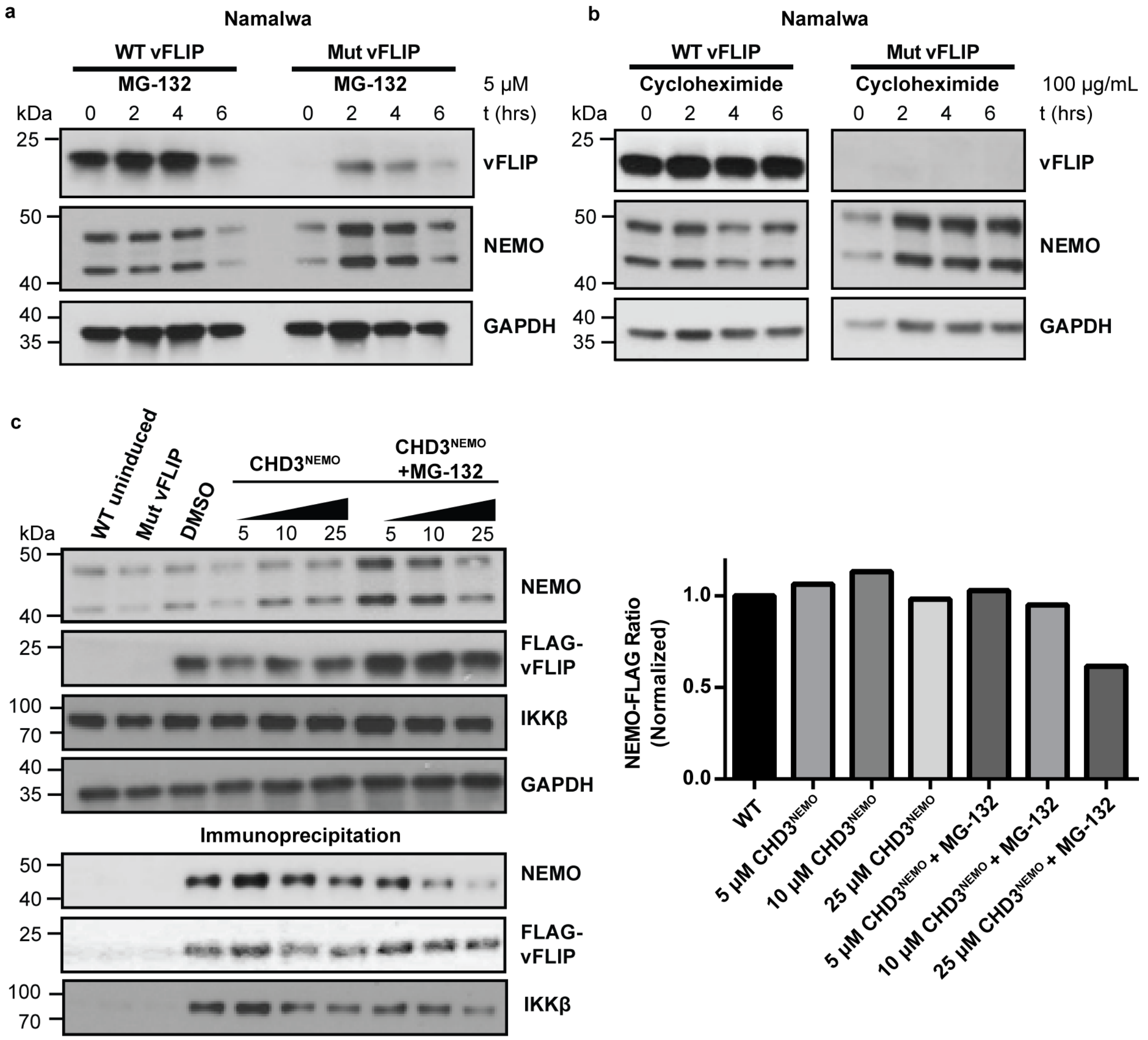

Supplementary Figure 10. vFLIP NF-kB dead mutant has a reduced half-life compared to WT vFLIP and MG-132 stabilizes its protein expression. Immunoblots of extracts of Namalwa WT vFLIP and mut vFLIP cell lines treated with (a) DMSO or 5 μM of the proteasomal inhibitor MG-132 for 0, 2, 4 and 6 hrs, (b) DMSO or 100 μg/ml of the protein biosynthesis inhibitor cycloheximide for 0, 2, 4 and 6 hrs. GAPDH was used as loading control. Data shown is a representative of three independent experiments (n=3).

(c) **CHD3^{NEMO}** treatment disrupts vFLIP/NEMO interaction and targets them for proteasomal degradation. Treating cells with **CHD3^{NEMO}** in the presence of the proteasomal inhibitor MG-132 can partially rescue vFLIP levels and reduce its binding to NEMO. Immunoblotting and co-immunoprecipitation assay of Namalwa WT vFLIP uninduced, mut vFLIP and WT vFLIP extracts treated with 0, 5,10 and 25 μM of **CHD3^{NEMO}** for 24 hrs in the presence or absence of 0.5 μM MG-132. Data shown is a representative of two independent experiments (n=2). Cell lysates were quantified using BCA assay and equal protein amounts were pulled down using FLAG M2 beads that recognize FLAG-tagged vFLIP followed by immunoblotting using NEMO and FLAG antibodies. Quantitation of the protein bands was performed using image J and the ratio of NEMO/vFLIP normalized to DMSO control in the immunoprecipitated lysates is displayed to the right.

Supplementary Table 4. List of a panel of lymphoma cell lines along with their viral status and sensitivity to the HSP90 inhibitor PU-H71 represented as LC_{50} in nanomolar concentration.[2,3]

**Cell line**	**Cell Type**	**PU-H71 LC_{50} [4]**	**Viral Status**
BC-1	PEL	36.9	$KSHV^+/EBV^+$
BC-2	PEL	27.1	$KSHV^+/EBV^+$
BC-3	PEL	63.4	$KSHV^+$
BCBL-1	PEL	217	$KSHV^+$
Namalwa	Burkitt	337.1	EBV^+

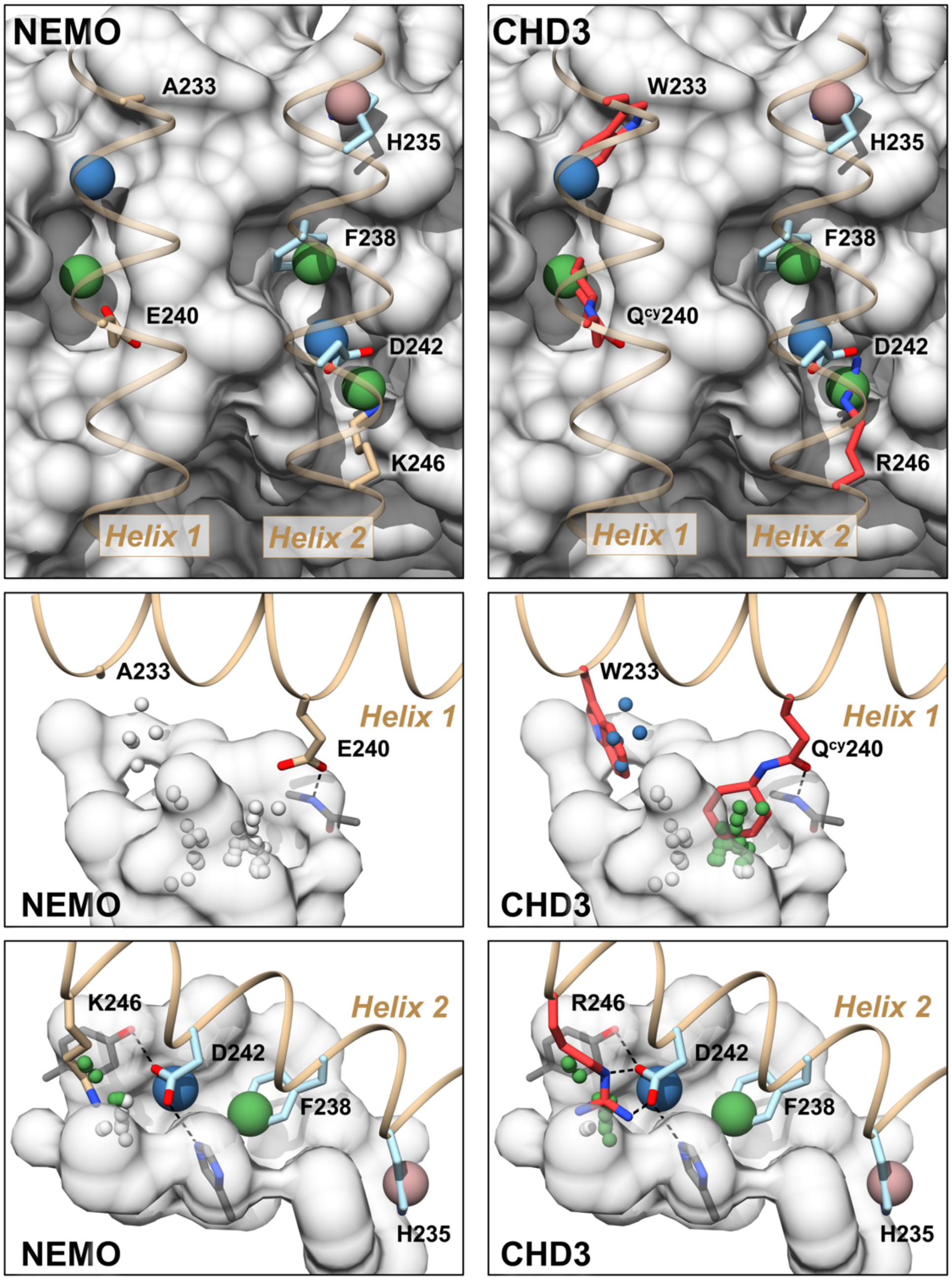

Supplementary Figure 11. AlphaSpace Design. Illustration of the 6 residue-centric pockets detected on the surface of vFLIP at the NEMO interface, comparing wild type NEMO residues (on the left) and optimized **CHD3^{NEMO}** residues (on the right). Residues are colored tan (NEMO wild type), red (**CHD3^{NEMO}** mutants), or light blue (unmodified). Pocket centroids are indicated by large spheres and colored green (alpha-space volume > 100 Å^3), blue (alpha-space volume > 50 Å^3), or pink (alpha-space volume > 10 Å^3). The alpha-space volume correlates well with a combination of pocket surface area and curvature. In lower panels, pockets targeted by optimization are shown as alpha clusters to highlight enhanced pocket occupancy in **CHD3^{NEMO}**; occupied alpha atoms are colored green/blue by pocket and unoccupied alpha atoms are colored white. Key polar interactions are highlighted by dashed black lines with participating vFLIP residues shown in dark grey.

Supplementary Table 5. List of pockets detected on the surface of vFLIP at the NEMO interface ranked by alpha-space volumes, along with associated NEMO/**CHD3^{NEMO}** residues, and % pocket occupancies.

Pocket	Binding Residue (NEMO > CHD3)	Alpha-Space Volume (Å^3)	% Occupancy (NEMO)	% Occupancy (CHD3)
**1**	Asp240 > Q^{cy}	251	0%	46%
**2**	Phe238	213	81%	
**3**	Lys246 > Arg	111	45%	75%
**4**	Asp242	58	30%	
**5**	Ala233 > Trp	52	0%	55%
**6**	His235	18	96%	

Compound Characterization

Supplementary Table 6. Mass spectroscopic analysis of synthetic peptides

Compound	Mass calculated, [M+H]$^+$	Mass observed, [M+H]$^+$
**UNC^{NEMO}**	2084.02	2084.36
**HBS^{NEMO}**	2122.03	2122.57
**CHD1^{NEMO}**	3840.12	3840.40
**CHD2^{NEMO}**	4020.24	4020.16
**CHD3^{NEMO}**	4101.33	4123.93
**CHD4^{NEMO}**	3920.26	3920.38
**FITC-CHD2^{NEMO}**	4436.28	4436.24
**FITC-CHD3^{NEMO}**	4517.38	4517.55

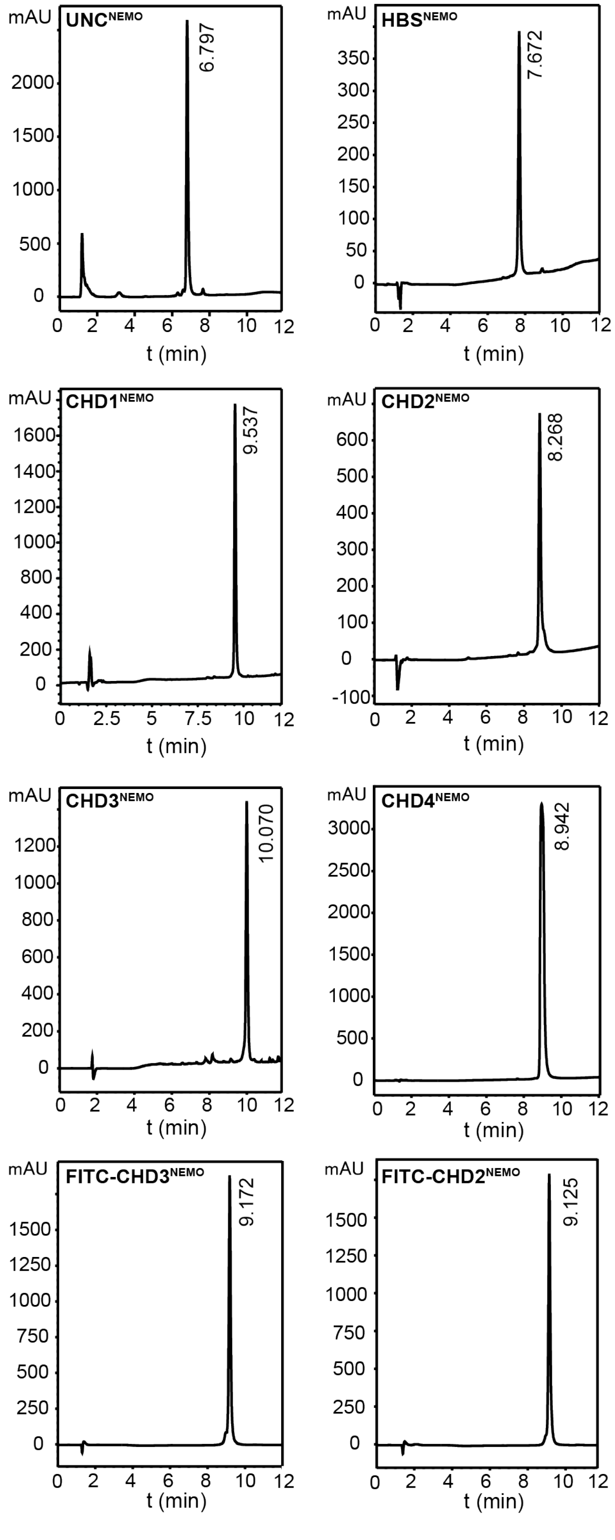

Supplementary Figure 12. Analytical HPLC traces of purified peptides

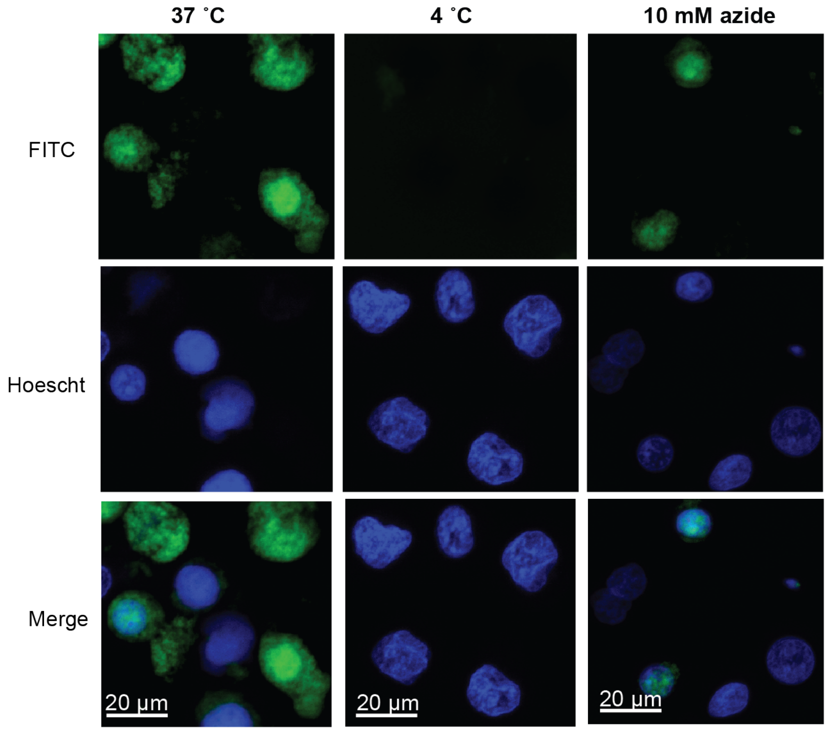

Supplementary Figure 13. Live confocal microscopy of FITC-CHD3^{NEMO}. Cellular uptake visualized after 1 hr incubation period. Effect of temperature and 10 mM sodium azide on cellular uptake. Images are representative of two independent experiments (n=2).

Supplementary References

1. Bagneris, C. et al. Crystal structure of a vFlip-IKKgamma complex: insights into viral activation of the IKK signalosome. *Mol Cell* **30**, 620-31 (2008).
2. Nayar, U. et al. Targeting the Hsp90-associated viral oncoproteome in gammaherpesvirus-associated malignancies. *Blood* **122**, 2837-47 (2013).
3. Giulino-Roth, L. et al. Inhibition of Hsp90 Suppresses PI3K/AKT/mTOR Signaling and Has Antitumor Activity in Burkitt Lymphoma. *Mol Cancer Ther* **16**, 1779-1790 (2017).
4. Shanmugam, M. et al. Targeting glucose consumption and autophagy in myeloma with the novel nucleoside analogue 8-aminoadenosine. *J Biol Chem* **284**, 26816-30 (2009).
