# Supplementary material for: Modulation of virus-induced NF-κB signaling by NEMO coiled coil mimics
Source: Nat Commun. 2020 Apr 14;11:1786. doi: 10.1038/s41467-020-15576-3 (PMC7156456; doi:10.1038/s41467-020-15576-3)
Supplement: Supplementary file 2 — Reporting Summary [file 41467_2020_15576_MOESM2_ESM.pdf]

nature research

Corresponding author(s): Ethel Cesarman and Paramjit Arora

Last updated by author(s): Februray 24 2020

Reporting Summary

Nature Research wishes to improve the reproducibility of the work that we publish. This form provides structure for consistency and transparency in reporting. For further information on Nature Research policies, see Authors & Referees and the Editorial Policy Checklist.

Statistics

For all statistical analyses, confirm that the following items are present in the figure legend, table legend, main text, or Methods section.

n/a	Confirmed	
☐	☒	The exact sample size (*n*) for each experimental group/condition, given as a discrete number and unit of measurement
☐	☒	A statement on whether measurements were taken from distinct samples or whether the same sample was measured repeatedly
☐	☒	The statistical test(s) used AND whether they are one- or two-sided<br>*Only common tests should be described solely by name; describe more complex techniques in the Methods section.*
☒	☐	A description of all covariates tested
☐	☒	A description of any assumptions or corrections, such as tests of normality and adjustment for multiple comparisons
☐	☒	A full description of the statistical parameters including central tendency (e.g. means) or other basic estimates (e.g. regression coefficient) AND variation (e.g. standard deviation) or associated estimates of uncertainty (e.g. confidence intervals)
☐	☒	For null hypothesis testing, the test statistic (e.g. *F*, *t*, *r*) with confidence intervals, effect sizes, degrees of freedom and *P* value noted<br>*Give P values as exact values whenever suitable.*
☒	☐	For Bayesian analysis, information on the choice of priors and Markov chain Monte Carlo settings
☒	☐	For hierarchical and complex designs, identification of the appropriate level for tests and full reporting of outcomes
☒	☐	Estimates of effect sizes (e.g. Cohen's *d*, Pearson's *r*), indicating how they were calculated

Our web collection on statistics for biologists contains articles on many of the points above.

Software and code

Policy information about availability of computer code

Data collection	TR-FRET data was collected using gen5 software , absorbance at 280nm was collected using FPLC system FPLC software, live confocal images were collected using ZEN microscope and imaging software, Flow cytometry data were collected using BD FACSDiVa™ software, bioluminescence images and quantification were collected using IVIS living image software
Data analysis	Microsoft excel 2010, Graphpad Prism version 6, FlowJo version 8.8.6, Fiji image processing package, Alphaspace 1.0 (http://www.nyu.edu/projects/yzhang/AlphaSpace/), Rosetta computational alanine scanning

For manuscripts utilizing custom algorithms or software that are central to the research but not yet described in published literature, software must be made available to editors/reviewers. We strongly encourage code deposition in a community repository (e.g. GitHub). See the Nature Research guidelines for submitting code & software for further information.

Data

Policy information about availability of data

All manuscripts must include a data availability statement. This statement should provide the following information, where applicable:

- Accession codes, unique identifiers, or web links for publicly available datasets
- A list of figures that have associated raw data
- A description of any restrictions on data availability

Data generated or analyzed during this study are included in the published article (and its supplementary information files) or are available from the corresponding author upon reasonable request, see author contributions for specific data sets.

Field-specific reporting

Please select the one below that is the best fit for your research. If you are not sure, read the appropriate sections before making your selection.

☒ Life sciences ☐ Behavioural & social sciences ☐ Ecological, evolutionary & environmental sciences

For a reference copy of the document with all sections, see nature.com/documents/nr-reporting-summary-flat.pdf

Life sciences study design

All studies must disclose on these points even when the disclosure is negative.

Sample size	No statistical method was used to determine sample size before experiments were performed. Sample size was calculated based on estomated effect. Dat represent two or three independent experiments each performed in replciates. Single experiments were performed for assay types such as protein expression and purification,size exclusion chromatography
Data exclusions	No data was excluded
Replication	Experiments were perfomed in duplicates and triplicates and repeated independently n=2 or n=3 as described in legend . Mice xenograft study was done once with n=10 control and n=5 peptide treated group
Randomization	Engrafted mice were randomized into vehicle and treated group. Each group had mice with similar low and high tumor burden to normalize for engraftment variability
Blinding	Data collection and analysis was not performed blindly

Reporting for specific materials, systems and methods

We require information from authors about some types of materials, experimental systems and methods used in many studies. Here, indicate whether each material, system or method listed is relevant to your study. If you are not sure if a list item applies to your research, read the appropriate section before selecting a response.

Materials & experimental systems

n/a	Involved in the study	
☐	☒	Antibodies
☐	☒	Eukaryotic cell lines
☒	☐	Palaeontology
☐	☒	Animals and other organisms
☒	☐	Human research participants
☒	☐	Clinical data

Methods

n/a	Involved in the study	
☒	☐	ChIP-seq
☐	☒	Flow cytometry
☒	☐	MRI-based neuroimaging

Antibodies

Antibodies used	The following primary antibodies were used: NEMO (1:500 or 1:1000, GTX107582, lot# 39946), GAPDH (1:25,000, GTX100118, lot #41577) from Genetex. phospho IKKα/ (Ser176/180) (1: 500, clone 16A6, cat #2697S,lot# 13), phospho p65 (Ser536)(1:500, clone 93H1,cat# 3033S ,lot #6), total p65 (1:500, clone C22B4, cat# 4764S, Lot# 8), phospho IkBα (S32/36) (1:500, cat# 9246S, lot#23), IkB (1:800, cat# 4814, lot #4), IKK (1:700, clone D30C6, cat #8943s, lot# 4) and HSP90 antibody (1:1000, 4877T) from cell signaling technology. IKKα (1:300, H-744, sc-7218, lot# 2903), NEMO (1:200, cat # sc-8330, lot# D0414) for pull down experiment, phospho p65 (1:200, sc-101748, lot D2809) for cell fractionation experiment and PCNA (1:500, cat # sc- 56, lot# E2814) were purchased from Santa Cruz Biotechnology. Anti-Flag (1:1000, cat# 600-401-383, lot# 28976) from Rockland. A rat monoclonal antibody to vFLIP (1:200, clone 4C1) was kindly provided by Elisabeth Kremmer at Helmholtz Zentrum Munchen, Germany. Secondary anti-HRP rabbit antibody (1:5000, NA9340V, GE healthcare), Secondary anti-HRP mouse antibody (1:2000, NA931V, GE healthcare) and goat anti-rat IgG (H&L) HRP antibody (1:5000, 62-9520, ThermoFisher Scientific). Rabbit Trueblot anti-Rabbit IgG HRP (1:1000, Rockland 18-8816-3, lot 30172) to enable detection of NEMO in the immunoprecipitated BC-1 cells without interference of the heavy or light chains.
Validation	Validation statement for each antibody is proivided on the manufracturer's website. vFlip antibody was validated in non-PEL cell lines that are not infected with KSHV so they don't express the viral protein vFLIP. Flag antibody was validated in Namalwa cell line that is not induced with doxycycline . This cell line doesn't express the inducible flag-tagged vFLIP protein unless doxycline was added

Eukaryotic cell lines

Policy information about cell lines

Cell line source(s)	BC1, BC2 and BC3 PEL cell lines were established in Dr.Cesarman laboratory from lymphomatous effusions as described previously (cesarman, 1995, arvanitakis, 1996) , BCBL-1 was obtained from the AIDS and Cancer Specimen Bank. Namalwa Burkitt lymphoma cell line was purchased from American Type Culture Collection (ATCC). Stable WT vFLIP and mutant vFLIP inducible Namalwa cell lines were generated from the parental Namalwa cell line by lentiviral transduction. BC3-NF-kb luc cell line was generated from BC3 cell line using plasmid selection. HeLa cells were purchased from ATCC (lot# 70016358)

Authentication	BC1, BC3, BC2, BCBL-1, Namalwa and HeLa cell lines were authenticated by short tandem repeat analysis (STR) profiling at IDEXX research
Mycoplasma contamination	All cell lines were tested negative for mycoplasma contamination using IDEXX Bioresearch services or in the lab using PCR Mycoplasma Detection Kit (abmGood)
Commonly misidentified lines (See ICLAC register)	no commonly misidentified cell lines were used

Animals and other organisms

Policy information about studies involving animals; ARRIVE guidelines recommended for reporting animal research

Laboratory animals	NOD.CB17-Prkdc (SCID) mice ordered from Jackson Laboratory
Wild animals	no wild animals were used for this study
Field-collected samples	the study did not include field-collected samples
Ethics oversight	mice studies were conducted according to IACUC protocol approved by Weill Cornell Medicine

Note that full information on the approval of the study protocol must also be provided in the manuscript.

Flow Cytometry

Plots

Confirm that:

☒ The axis labels state the marker and fluorochrome used (e.g. CD4-FITC).

☒ The axis scales are clearly visible. Include numbers along axes only for bottom left plot of group (a 'group' is an analysis of identical markers).

☒ All plots are contour plots with outliers or pseudocolor plots.

☒ A numerical value for number of cells or percentage (with statistics) is provided.

Methodology

Sample preparation	Details are outlined in materials and methods section. BC-1 PEL cells were treated with DMSO or increasing concentrations of the CHD3NEMO or CHD4NEMO peptide for one hour in the absence of serum then 20% FBS was added 1hr after serum starvation. Cells were harvested at 48 hrs post-treatment, washed once in PBS and resuspended in Annexin V staining buffer (BD Pharmingen Catalog No. 556454) containing 3 µL/test AnnexinV-Alexa Fluor 647 (ThermoFisher A23204) and 1 uL/test DAPI (Sigma D9542) and incubated at room temperature for 15 minutes in the dark. (Single stain compensation controls were used: unstained cells, DAPI only and Alexa647 only)
Instrument	BD LSRII analytical flow cytometer
Software	Flow cytometry data were collected using BD FACSDiVa™ software and data was analyzed using FlowJo version 8.8.6
Cell population abundancea	0.5 million cells were stained for each condition and 300,000 to 500,000 events were collected from control and treated cells
Gating strategy	A representative density plot with set gates is illustrated in figure 5C. Briefly, histogram with single stain compensation controls were used to distinguish positive and negative stains. Total population (live and dead cells) were gated on after excluding cellular debris based on their FSC-SSC distribution. Bivariate pseudocolor plot (Annexin-Alexa647, DAPI-Pacific blue) using single stain compensation controls was used to set the gates and the same gate was applied to all sample conditions.

☐ Tick this box to confirm that a figure exemplifying the gating strategy is provided in the Supplementary Information.
