# Supplementary material for: Modulation of virus-induced NF-κB signaling by NEMO coiled coil mimics
Source: Nat Commun. 2020 Apr 14;11:1786. doi: 10.1038/s41467-020-15576-3 (PMC7156456; doi:10.1038/s41467-020-15576-3)
Supplement: Supplementary file 3 — Source Data [file 41467_2020_15576_MOESM3_ESM.pdf]

Full blots corresponding to each Figure

Figure 4. (c) CHD3[NEMO] disrupts vFLIP/NEMO interaction in live cells.

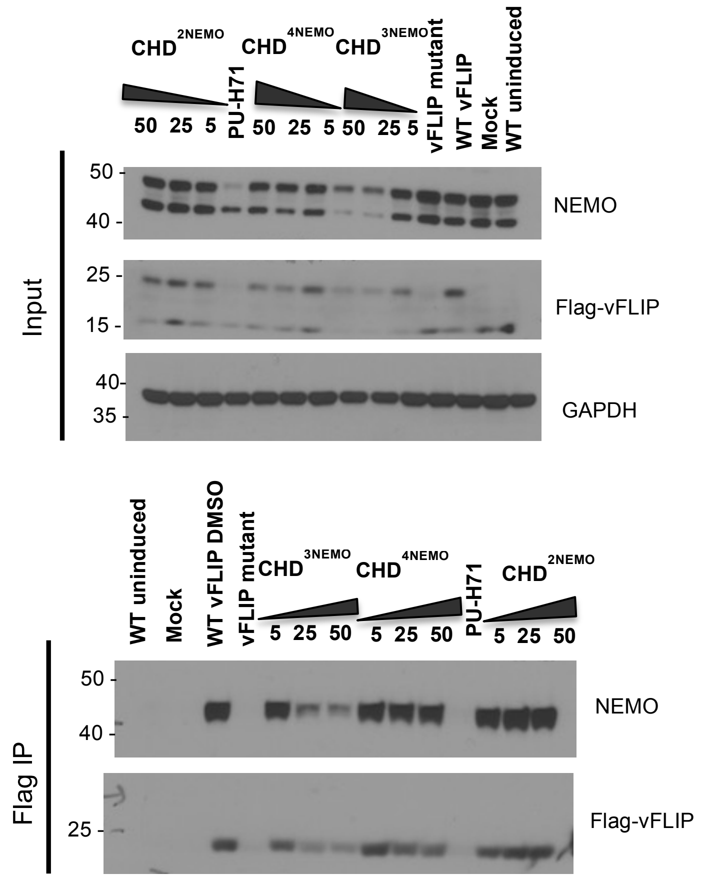

Figure 6 (d) Mice treated with **CHD3^{NEMO}** for 24hrs have attenuated NF-κB signaling in tumor cells.

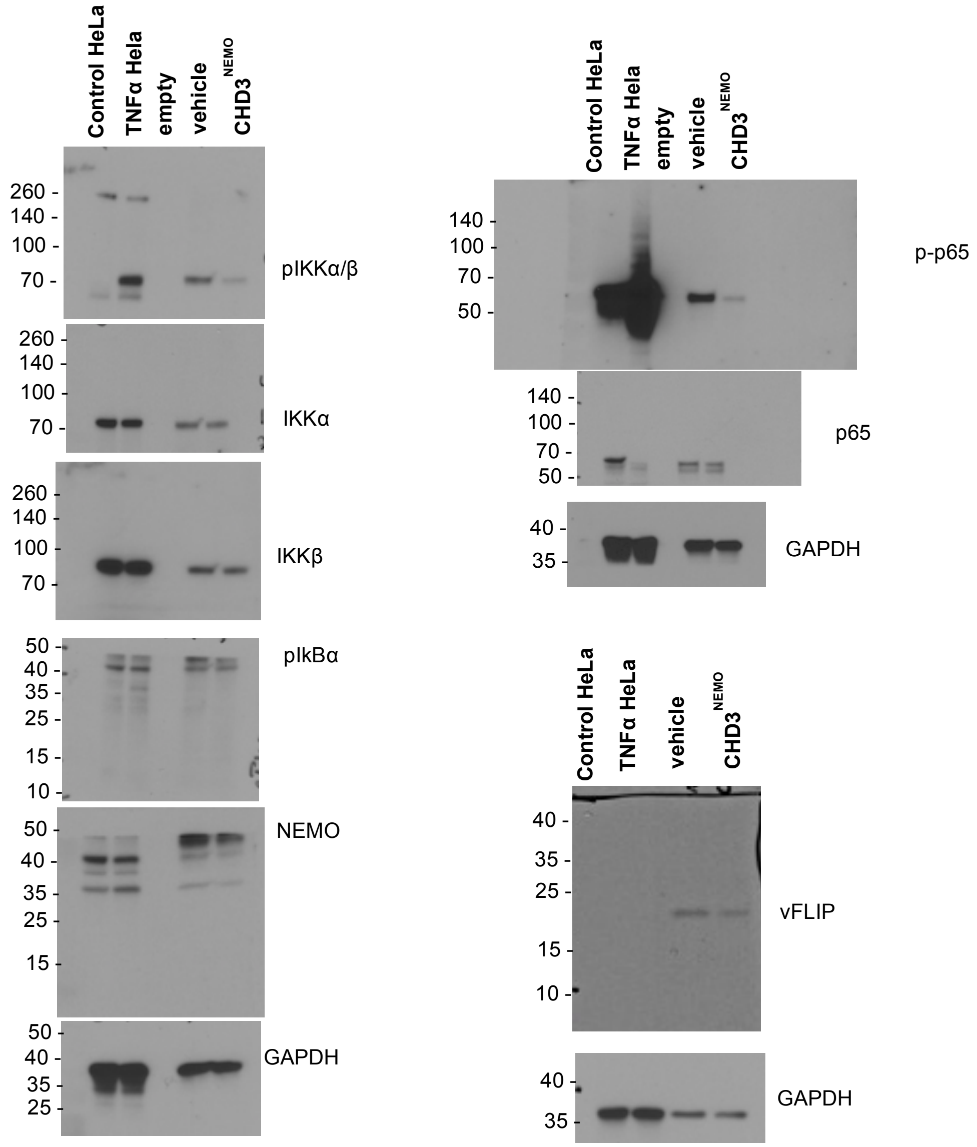

Supplementary Figure 5. CHD3^{NEMO} inhibits NF-κB signaling in BC1 PEL cell line.

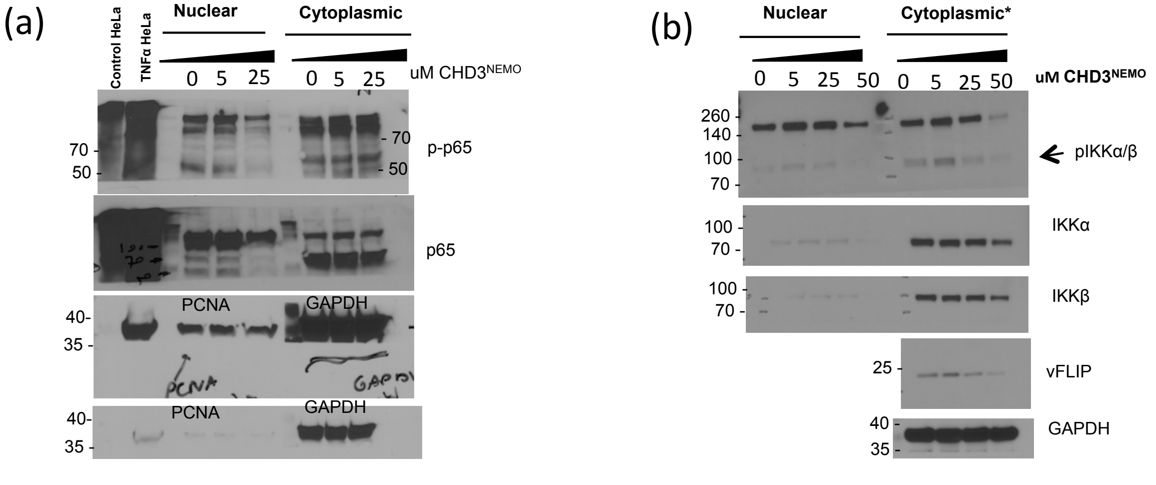

* Cytoplasmic fractions in paper

Supplementary Figure 6. CHD3^{NEMO} pretreatment does not block TNFα stimulation and NF-κB signaling in HeLa cells.

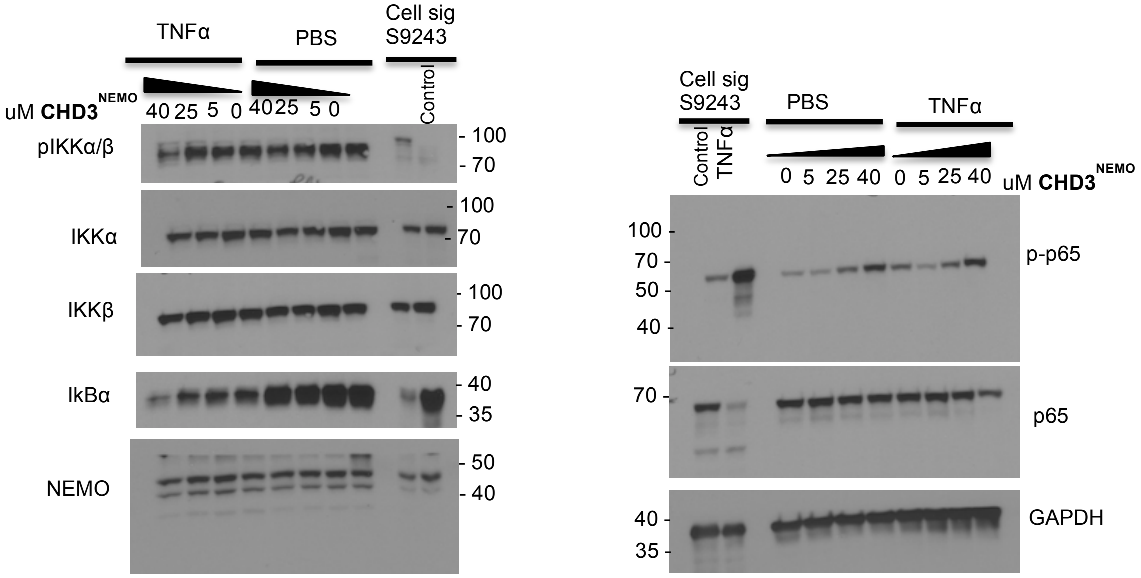

Supplementary Figure 7. CHD3^{NEMO} treatment downregulates vFLIP target expression in BC-1 PEL cells

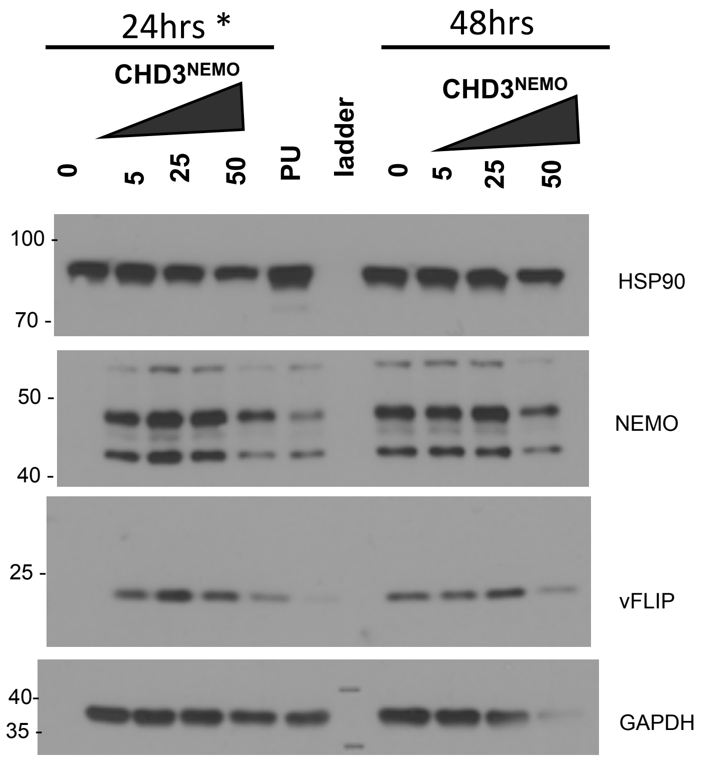

*24hr Included in paper

Supplementary Figure 8. CHD3^{NEMO} treatment decreases vFLIP association with NEMO in BC-1 PEL cells

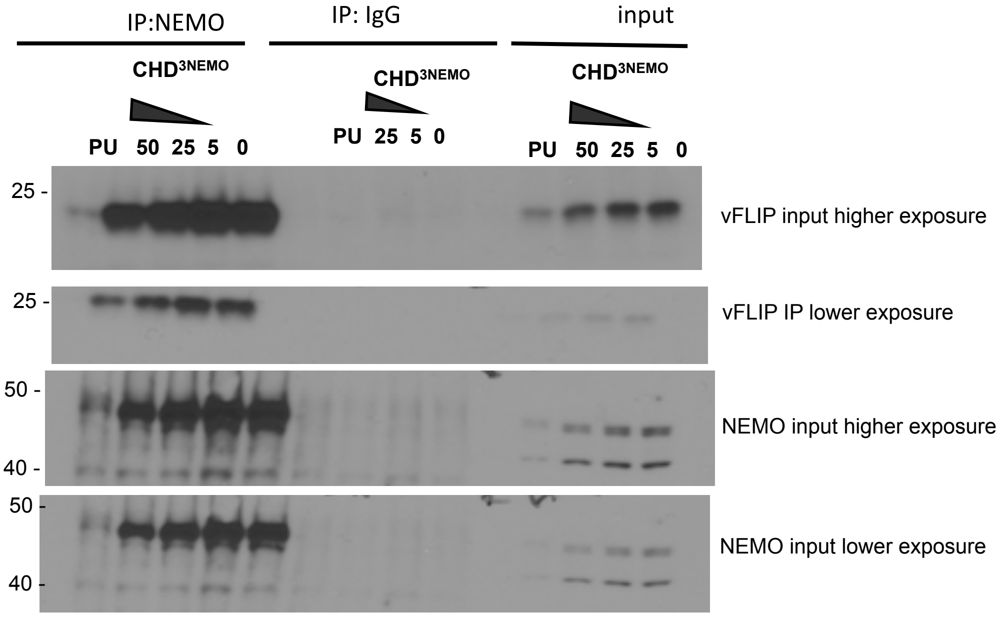

Supplementary Figure 9. Effect of CHD3^{NEMO} on NEMO levels in Namalwa cells expressing vFLIP target

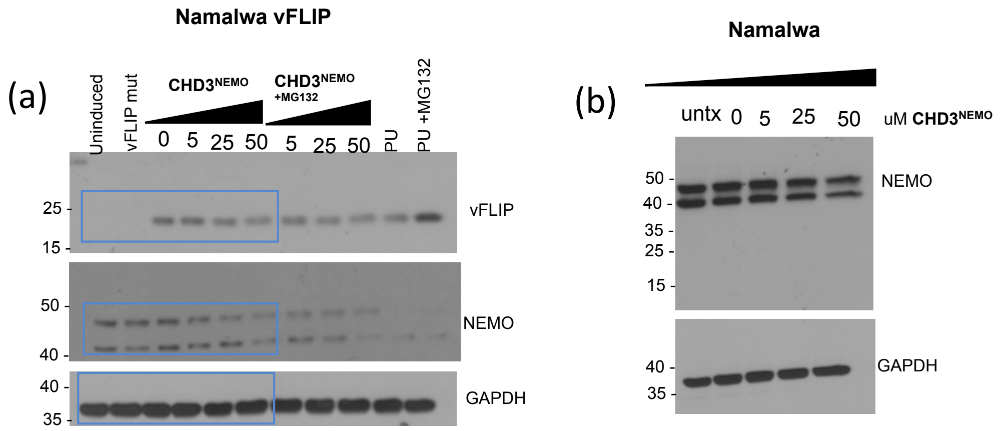

Supplementary Figure 10. (a) and (b) vFLIP NF-kB dead mutant has a reduced half-life compared to WT vFLIP and MG-132 stabilizes its protein expression. (c)) **CHD3^{NEMO}** treatment disrupts vFLIP/NEMO interaction and targets them for proteasomal degradation

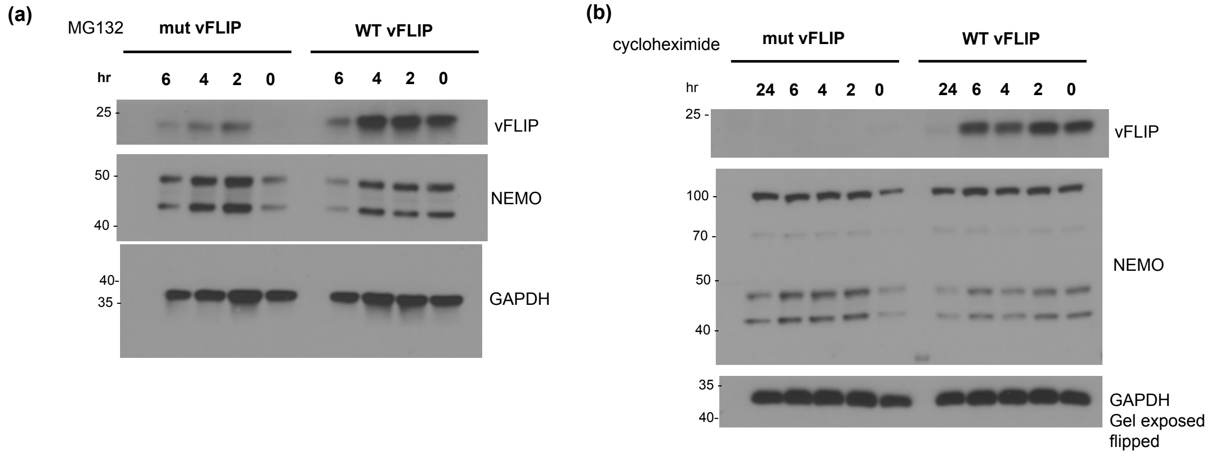

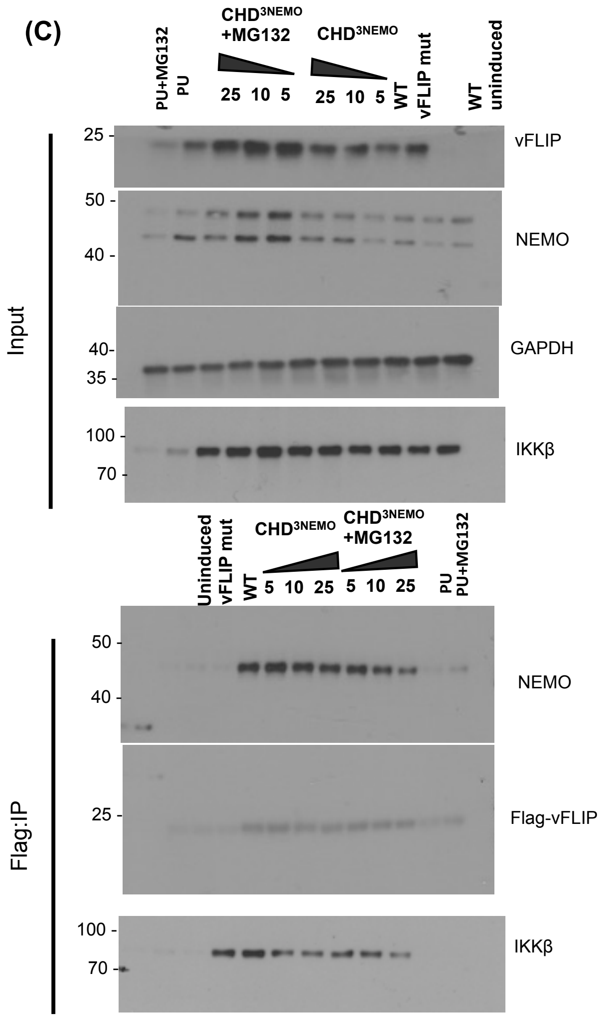
(C)
PU+MG132
PU
CHD3NEMO
+MG132
25 10 5
CHD3NEMO
25 10 5
WT
vFLIP mut
WT
uninduced
Input
vFLIP
NEMO
GAPDH
IKKβ
Uninduced
vFLIP mut
WT
CHD3NEMO
5 10 25
CHD3NEMO
+MG132
5 10 25
PU
PU+MG132
Flag:IP
NEMO
Flag-vFLIP
IKKβ
